# Engineered MSC‐sEVs as a Versatile Nanoplatform for Enhanced Osteoarthritis Treatment via Targeted Elimination of Senescent Chondrocytes and Maintenance of Cartilage Matrix Metabolic Homeostasis

**DOI:** 10.1002/advs.202413759

**Published:** 2025-01-04

**Authors:** Kai Feng, Jiashuo Liu, Liangzhi Gong, Teng Ye, Zhengsheng Chen, Yang Wang, Qing Li, Xuetao Xie

**Affiliations:** ^1^ Institute of Microsurgery on Extremities Department of Orthopedic Surgery Shanghai Sixth People's Hospital Affiliated to Shanghai Jiao Tong University School of Medicine Shanghai 200233 China

**Keywords:** engineered modification, MSC‐sEVs, osteoarthritis, senescent chondrocytes, targeted therapy

## Abstract

Chondrocyte senescence is an important pathogenic factor causing osteoarthritis (OA) progression through persistently producing pro‐inflammatory factors. Mesenchymal stem cells‐derived small extracellular vesicles (MSC‐sEVs) have shown anti‐inflammatory effects in OA models, while persistent existence of senescent chondrocytes still promotes cartilage destruction. Therefore, improving the targeted elimination ability on senescent chondrocytes is required to facilitate the translation of MSC‐sEVs in OA treatment. In this study, versatile engineered MSC‐sEVs are developed to targetedly clear senescent chondrocytes and maintain cartilage metabolic homeostasis. Specifically, MSC‐sEVs are loaded with siRNA mouse double minute 2 homologue (siMDM2) and modified with cartilage‐targeting peptide WYRGRL‐PEG_2K_‐DSPE (WPD), named WPD‐sEVs^siMDM2^. The results demonstrate versatile modification improves the cellular uptake of MSC‐sEVs in chondrocytes, and thus improves the antiaging effects. Importantly, multifunctional modification enhances cartilage penetration ability and extends joint retention time of MSC‐sEVs. In both post‐traumatic OA mice and naturally aged mice, WPD‐sEVs^siMDM2^ more effectively eliminates senescent chondrocytes and maintained matrix metabolic homeostasis. By using the P53 phosphorylation inhibitor, the essential role MDM2‐P53 pathway in the antiaging function of WPD‐sEVs^siMDM2^ on chondrocytes is verified. In ex vivo cultured human OA cartilage explants, it is confirmed that WPD‐sEVs^siMDM2^ alleviates senescent phenotype. Altogether, the findings suggest that WPD‐sEVs^siMDM2^ have promising translational potential for OA treatment.

## Graphical abstract

Multifunctionally engineered WPD‐sEVs^siMDM2^ targets the articular cartilage in OA joints after intra‐articular injection and efficiently penetrates into the deep zone of cartilage. Subsequently, WPD‐sEVs^siMDM2^ successfully reverses senescent phenotype by delivering siMDM2 and other bioactive molecules in the original MSC‐sEVs. Specifically, WPD‐sEVs^siMDM2^ effectively eliminates senescent chondrocytes and inhibits SASP factors secretion, thus maintaining cartilage ECM homeostasis and alleviating OA progression. The current findings open new avenues for the development of sEVs‐based therapies for the treatment of OA and other age‐related diseases.

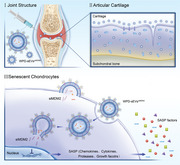

## 1 Introduction

Chondrocyte senescence is the essential pathogenic mechanism that can both promote the progression of age‐associated osteoarthritis (OA) and post‐traumatic OA (PTOA).^[^ ^1^ ^]^ During OA development, the healthy phenotype of chondrocytes that maintains the cartilage matrix metabolic homeostasis is lost gradually.^[^ ^2^ ^]^ Instead, it is switched to senescent phenotype, leading to the erosion of extracellular matrix including collagen II network and proteoglycan through secreting excessive senescence‐associated secretory phenotype (SASP) factors including pro‐inflammatory cytokines, chemokines and enzymes.^[^ ^1^, ^2^ ^]^ Specifically, excessive and persistent loading of articular cartilage caused by aging and traumatic stress lead to the release of SASP factors in chondrocytes, which can promote chronic inflammation and induce cellular senescence through up‐regulating P53.^[^ ^1^ ^]^ When P53 phosphorylation and its activity is inhibited, senescent cells do not undergo apoptosis and accumulate in aged tissues, which cause surrounding cells dysfunction and structure damage.^[^ ^3^ ^]^ Previous studies showed mouse double minute 2 homologue (MDM2)‐P53 pathway plays an important role in the apoptosis of senescent cells. In specific, MDM2 can bind with P53 and inhibit P53 phosphorylation and its activity, thus suppress the apoptosis of senescent cells.^[^ ^4^ ^]^ Therefore, manipulating and clearing senescent chondrocytes is essential to develop available disease‐modifying strategy for OA treatment.

Recent studies have demonstrated that mesenchymal stem cells‐derived small extracellular vesicles (MSC‐sEVs)‐based cell‐free therapy is a promising strategy for OA treatment.^[^ ^5^ ^]^ In particular, MSC‐sEVs can deliver abundant bioactive nucleic acids and proteins, thus attenuate joint inflammatory responses and inhibit cartilage matrix catabolism.^[^ ^6^ ^]^ However, MSC‐sEVs are insufficient for eliminating senescent chondrocytes in OA cartilage radically as these accumulated senescent chondrocytes can continuously secrete pro‐inflammatory SASP factors to promote OA progression. Thus, there is an urgent need to develop a novel strategy for enhancing the therapeutic function of MSC‐sEVs, especially on senescent chondrocytes. MSC‐sEVs‐based RNA interference (RNAi) technology is an effective approach to improve the therapeutic effects of MSC‐sEVs.^[^ ^7^ ^]^ As natural vesicles secreted by parental MSCs, MSC‐sEVs have low immune rejection, natural affinity, and high tissue permeability,^[^ ^8^ ^]^ making MSC‐sEVs an ideal nanoplatform to construct the delivery system and realize the stable delivery of siRNA into cells for improving the therapeutic function. The siRNA‐loaded MSC‐sEVs can not only take advantage of the intrinsic anti‐inflammatory function of MSC‐sEVs but also collaborate with the loaded siRNA to enhance the therapeutic effects. As such, MSC‐sEVs‐based siRNA delivery nanoplatform can be developed to clear senescent chondrocytes and improve the antiaging effects of MSC‐sEVs for OA treatment.

Using MSC‐sEVs as the siRNA delivery nanoplatform helped to enhance the therapeutic function of MSC‐sEVs, but it is not sufficient to realize targeted delivery and treatment of OA cartilage and senescent chondrocytes. Currently, direct intra‐articular injection is the main route for MSC‐sEVs delivery.^[^ ^9^ ^]^ However, nonspecific biodistribution of MSC‐sEVs in the joint severely limit the therapeutic efficacy, as the joint is a complex structure which consists of articular cartilage, synovium, subchondral bone, meniscus, ligaments.^[^ ^9^ ^]^ Several studies have demonstrated targeting the component of cartilage might provide an effective avenue for efficient OA treatment.^[^ ^10^ ^]^ Targeting collagen II has been explored for enhancing specificity to articular cartilage and extending retention time in the joint cavity. It was reported collagen II‐targeting sequence WYRGRL led to significant increase (≈72‐fold) of cartilage‐targeting efficiency for the peptide‐engineered nanoplatform.^[^ ^11^ ^]^ Formica et al. found that WYRGRL‐conjugated dexamethasone showed extended retention time in the cartilage and enhanced therapeutic efficacy compared to free dexamethasone.^[^ ^12^ ^]^ In another study, WYRGRL peptide was conjugated on the surface of metal organic framework‐decorated mesoporous polydopamine to construct a cartilage‐targeting delivery nanoplatform. This nanoplatform exerted potent antiapoptosis and anti‐inflammation effects and alleviated cartilage degeneration in OA rat.^[^ ^10a^ ^]^

On the other hand, delivery of MSC‐sEVs to senescent chondrocytes is particularly challenging because MSC‐sEVs have to penetrate through a dense layer of articular cartilage composed of a complex meshwork of collagen II fibrils (50–60% dry weight) densely packed with highly negatively charged proteoglycan (35% dry weight).^[^ ^13^ ^]^ Since a majority of chondrocytes reside in the middle and deep zone of articular cartilage,^[^ ^13^ ^]^ efficient delivery of bioactive factors and siRNA in MSC‐sEVs to senescent chondrocytes remains challenging because of electrostatic hinderance. Recent studies proposed that surface charge‐based interaction between cationic and anionic articular cartilage is the key for efficient penetration.^[^ ^9^, ^14^ ^]^ Our previous study has shown that cationic amphiphilic macromolecule ε‐polylysine‐polyethylene‐distearyl phosphatidylethanolamine (PPD) engineered modification effectively reversed the negative surface charge of MSC‐sEVs. Intra‐articular injection of PPD engineered MSC‐sEVs exhibited improved bioavailability and enhanced therapeutic efficacy with reduced injection frequency in contrast to unmodified MSC‐sEVs.^[^ ^15^ ^]^ Therefore, MSC‐sEVs‐based delivery nanoplatform that capable of targeting articular cartilage and avoiding electrostatic hinderance after intra‐articular injection should be further optimized to substantially improve the bioavailability and minimize the off‐target effect.

**Figure 1:**
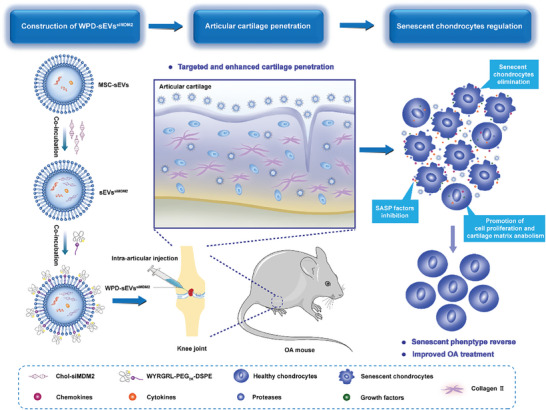
Schematic illustration showing the construction process of and mechanism of multifunctionally engineered WPD‐sEVs^siMDM2^ with enhanced cartilage penetration ability and targeted senescent chondrocytes elimination effect for OA treatment. Intra‐articular injection of WPD‐sEVs^siMDM2^ successfully reduced the percentage of senescent chondrocytes, inhibited SASP factors production, and retarded cartilage degeneration in OA mice.

In light of the above, in addition to enabling targeted delivery to cartilage efficiently, an effective siRNA which can improve the antiaging effects in OA cartilage should be encapsuled into MSC‐sEVs to clear senescent chondrocytes and maintain cartilage matrix metabolic homeostasis. Based on the important role of MDM2‐mediated apoptosis inhibition on senescent cells accumulation, siRNA MDM2 (siMDM2) was synthesized and loaded into MSC‐sEVs in this study. To further overcome the electrostatic hinderance and nonspecific biodistribution of MSC‐sEVs in the joint space, a novel collagen II‐targeting cationic peptide WYRGRL‐PEG_2K_‐DSPE (WPD) was developed to modify MSC‐sEVs. After serial ultracentrifugation, multifunctionally engineered MSC‐sEVs (WPD‐sEVs^siMDM2^) were obtained and this nanoplatform exhibited enhanced cellular uptake efficiency, improved cartilage penetration ability, and extended retention time in the joint. Our results highlight the ability of WPD‐sEVs^siMDM2^ to decrease the percentage of senescent chondrocytes and maintain matrix metabolic homeostasis in vitro and in vivo. In senescent human chondrocytes, we found WPD‐sEVs^siMDM2^ treatment significantly reduced the number of senescent cells and SASP factors secretion. In both PTOA mice and naturally aged mice, our results showed WPD‐sEVs^siMDM2^ treatment substantially downregulated the number of senescent cells and inhibited matrix catabolism. Moreover, we confirmed the key role MDM2‐P53 pathway in the antiaging function of WPD‐sEVs^siMDM2^ on chondrocytes by inducing apoptosis of senescent cells. Additionally, we verified the enhanced antiaging effects and protective effects of multifunctionally engineered MSC‐sEVs in ex vivo cultured human OA cartilage explants. Overall, our results indicated that this versatile engineered MSC‐sEVs‐based nanoplatform offers great promise for targeted OA therapy (**Figure** **1**).

## 2 Results

### 2.1 Preparation and Characterization of the Versatile Vesicles, WPD‐sEVs^siMDM2^

**Figure 2:**
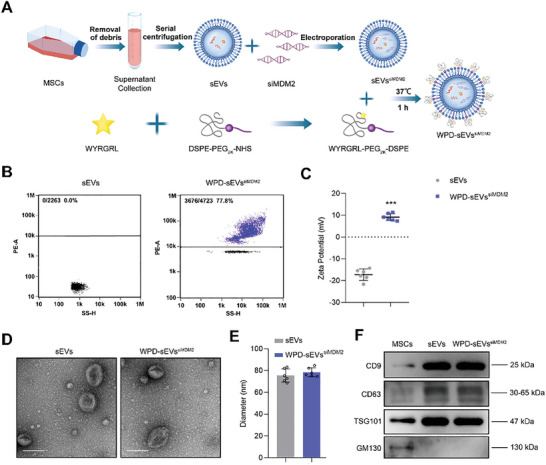
Construction and characterization of WPD‐sEVs^siMDM2^. A) Schematic diagram of the multifunctionally engineered MSC‐sEVs delivery system for the treatment of OA. B) Nanoflow cytometry (NFC) analysis of the WPD modification rate of sEVs incubating with 100 µg ml^−1^ Rhodamine B labeled WPD suspension. Rhodamine B labeled WPD (100 µg ml^−1^) was incubated with sEVs^siMDM2^, and WPD‐sEVs^siMDM2^ was isolated by ultracentrifugation. C) Zeta potential analysis of sEVs and WPD‐sEVs^siMDM2^ (*n* = 6 biologically independent samples). D) Transmission electron microscope (TEM) images of sEVs and WPD‐sEVs^siMDM2^. Scale bar: 100 nm. E) The average diameter of unmodified sEVs and WPD‐sEVs^siMDM2^ was detected by NFC analysis (*n* = 6 biologically independent samples). F) Western blot analysis of the expressions of surface protein markers including CD9, CD63, and TSG101. Data are represented as mean ± SD. ***P < 0.001.

First, MSCs were successfully derived from iPSCs using our induction protocol and flow cytometry analysis results showed that the MSCs highly expressed CD29, CD44, CD73, CD90, and CD105, and were negative for CD45, HLA‐DR, and CD34 (Figure S1, Supporting Information). To prepare WPD‐sEVs^siMDM2^, MSC‐sEVs were isolated from serum‐free culture medium of MSCs by the ultra‐centrifugation method, and siMDM2 was loaded into sEVs to construct sEVs^siMDM2^ by using the electroporation technology. Nanoflow cytometry (NFC) analysis demonstrated efficient siMDM2 loading (25.80% ± 1.16%) into sEVs (Figure S2, Supporting Information). Next, WYRGRL‐PEG_2K_‐DSPE (WPD) was synthesized by the amide ligation between WYRGRL and DSPE‐PEG_2K_‐NHS, and then incubated with sEVs^siMDM2^ to obtain multifunctionally engineered WPD‐sEVs^siMDM2^ (**Figure** **2**A). MOLDI‐TOF analysis showed the molecular weight of WPD was about 3.5 KDa (Figure S3A, Supporting Information). WPD was cytocompatible as there was no difference between the proliferation of human chondrocytes cultured with medium containing WPD at the concentration range of 0–100 µg ml^−1^ (Figure S3B,C, Supporting Information), thus, 100 µg ml^−1^ WPD was used to incubate with sEVs. To verify the successful modification of sEVs, the WPD peptide was labeled with rhodamine B. NFC analysis showed that 100 µg ml^−1^ cartilage‐targeting peptide WPD was successfully anchored onto the surface of sEVs (Figure 2B). Furthermore, to confirm the possible contaminant of soluble WPD or aggregating WPD, 100 µg ml^−1^ rhodamine B labeled WPD was treated as the procedure for WPD‐sEVs^siMDM2^ isolation. After ultracentrifugation, no fluorescent signal was detected by NFC analysis in the resuspension solution that rinsed the tube bottom, further excluding the possibility of soluble or aggregating WPD contaminant during WPD‐sEVs^siMDM2^ isolation. After the successful modification to sEVs, the zeta potential of WPD‐sEVs^siMDM2^ was analyzed. The average zeta potential of the unmodified sEVs was ‐17.23 ± 2.42 mV, while WPD‐sEVs^siMDM2^ presented an average zeta potential of 9.13 ± 1.31 mV, demonstrating the successful surface charge reverse of sEVs (Figure 2C). Transmission electron microscope (TEM) observation revealed that both sEVs and WPD‐sEVs^siMDM2^ were double‐layer vesicles with typical cup shape and intact membrane structure (Figure 2D). Additionally, no signs of sEVs aggregation or WPD aggregating contaminant were observed in the TEM image of WPD‐sEVs^siMDM2^. The distribution of particle size was detected by NFC analysis and the average diameter of unmodified sEVs and WPD‐sEVs^siMDM2^ were 77.17 ± 4.24 nm and 78.47 ± 3.76 nm, respectively (Figure 2E). Western blot analysis showed that both sEVs and WPD‐sEVs^siMDM2^ expressed the specific surface protein makers CD9, CD63, and TSG101, but did not express GM130 (Figure 2F), which well met the identification criteria for sEVs.

Physiological stability of WPD modification is of significant importance in the application of siMDM2 delivery to senescent chondrocytes in joint cavity, thus we evaluated the WPD modification rate, zeta potential, and average size of WPD‐sEVs^siMDM2^ in different solutions for a 7‐day period. First, the modification rate, zeta potential, and average diameter of WPD‐sEVs^siMDM2^ presented no notable difference after 7 days incubation in phosphate‐buffered saline (PBS) solution (Figure S4, Supporting Information), which indicates WPD‐sEVs^siMDM2^ presented an excellent stability in PBS solution. As anionic biomacromolecules hyaluronic acid (HA) and chondroitin sulfate (CS) distribute widely in the joint cavity, and anionic HA or CS can compete with the sEVs to bind WPD by electrostatic interaction, causing the detachment of WPD from sEVs surface and final modification failure. Therefore, the WPD modification stability under the presence of HA or CS was further evaluated by detecting the WPD modification rate. We found only a slightly decrease in modification rate was detected in WPD‐sEVs^siMDM2^ incubated with HA and CS solution for 7 days, as about 65% WPD modification rate was kept for WPD‐sEVs^siMDM2^ after incubation with HA (65.13% ± 0.57%) or CS (63.83% ± 1.13%) for 7 days (Figure S5, Table S1, Supporting Information). Altogether, these results verified a successful self‐assembly of WPD on sEVs lipid membrane and a relatively stable WPD modification to the lipid membrane of sEVs under mimic physiological environment of the joint.

### 2.2 Protective Effects of WPD‐sEVs^siMDM2^ on Senescent Phenotype and Matrix Metabolism in Human Chondrocytes

**Figure 3:**
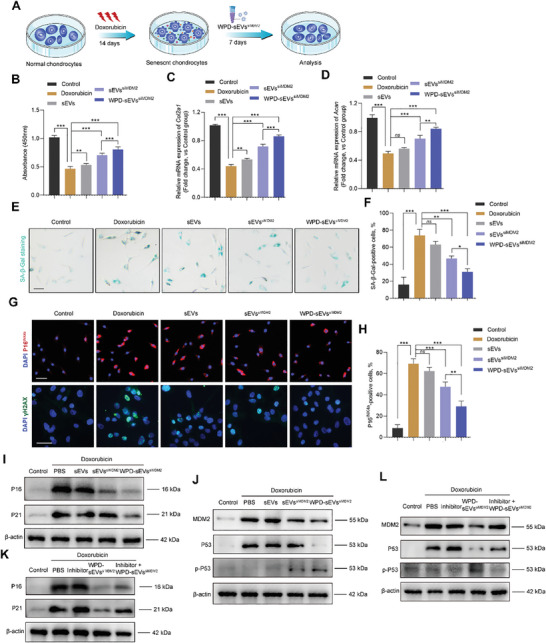
Effects of WPD‐sEVs^siMDM2^ on senescent phenotype and matrix metabolism homeostasis in doxorubicin‐induced human chondrocytes. A) Schematic diagram depicting the experimental timeline of doxorubicin induction (1 µM) that elicits inflammatory responses and senescent phenotype, treatment with 1 × 10^10^ particles ml^−1^ WPD‐sEVs^siMDM2^ for 7 days and subsequent analysis to evaluate senescent phenotypes in chondrocytes. B) The proliferative ability of chondrocytes was detected by CCK8 assay after different treatments (*n* = 6). C,D) PCR analysis of cartilage matrix anabolism‐related genes (*Col2a1* and *Acan*) in chondrocytes (*n* = 3). E) SA‐β‐Gal staining was performed after different treatments. Scale bar: 100 µm. F) Statistical evaluation of SA‐β‐Gal‐positive rate in chondrocytes (*n* = 3). G) Immunofluorescence staining for senescence marker P16^INK4a^ (red color) and γH2AX (green color) in chondrocytes. Scale bar: 100 µm. H) Statistical evaluation of P16^INK4a^‐positive rate in chondrocytes (*n* = 3). I) Western blot analysis for the protein expression of senescence markers P16^INK4a^ and P21 after different treatments. J) Western blot analysis for the protein expression of MDM2, P53 and p‐P53 after different treatments. K) Western blot analysis for the protein expression of P16^INK4a^ and P21 after the treatment of P53 phosphorylation inhibitor. L) Western blot analysis for the protein expression of MDM2, P53 and p‐P53 after the treatment of P53 phosphorylation inhibitor. Data are represented as mean ± SD. *P < 0.05, **P < 0.01, and ***P < 0.001, *ns*, not significant.

As senescent chondrocytes accumulation plays essential role in the progression of OA, we sought to determine whether versatile modification could enhance the antiaging effects of sEVs in vitro, and thus a DNA‐damaging chemical agent doxorubicin was applied in human chondrocytes to induce senescent phenotype. First, we determined the optimal concentration of doxorubicin in inducing chondrocyte senescence and we found doxorubicin presented no obvious cytotoxic effects on chondrocytes at the concentration of 0–1 µM (Figure S6, Supporting Information). Thus, doxorubicin at the concentration of 1 µM was used in our following experiments. To evaluate the antiaging effects of WPD‐sEVs^siMDM2^ in vitro, doxorubicin‐induced chondrocytes were co‐treated with sEVs (1 × 10^10^ particles ml^−1^), sEVs^siMDM2^ (1 × 10^10^ particles ml^−1^), or WPD‐sEVs^siMDM2^ (1 × 10^10^ particles ml^−1^) for 7 days (**Figure** **3**A). Then we assessed the cellular uptake of WPD‐sEVs^siMDM2^ in human chondrocytes. Our results showed that WPD‐sEVs^siMDM2^ displayed a better cellular uptake capacity than unmodified sEVs (Figure S7A, Supporting Information). Flow cytometry analysis further demonstrated a higher mean fluorescence intensity (MFI) of WPD‐sEVs^siMDM2^‐treated chondrocytes in comparison with that of unmodified sEVs‐treated chondrocytes (Figure S7B, Supporting Information), suggesting an enhanced chondrocyte uptake ability of WPD‐sEVs^siMDM2^. As the improved cellular uptake efficiency might promote the protective effects, then we further evaluated the senescent phenotype and cartilage matrix metabolic homeostasis in doxorubicin‐induced chondrocytes after WPD‐sEVs^siMDM2^ treatment. Our results showed the absorbance in WPD‐sEVs^siMDM2^‐treated senescent chondrocytes was significantly higher than that of sEVs or sEVs^siMDM2^ treatment group (Figure 3B), indicating an enhanced effect of multifunctionally engineered sEVs on chondrocyte proliferation. We next investigated the effect of WPD‐sEVs^siMDM2^ treatment on matrix anabolism in senescent chondrocytes, WPD‐sEVs^siMDM2^ treatment more effectively promoted the mRNA expressions of *Col2a1* (Figure 3C) and *Acan* (Figure 3D), as compared to the other treatment groups, further validating the enhanced antiaging effects of WPD‐sEVs^siMDM2^. Then, the percentage of doxorubicin‐induced senescent chondrocytes after different treatments was detected by senescence‐associated β‐galactosidase (SA‐β‐Gal) staining. Our results showed doxorubicin‐induced senescent chondrocytes (73.78% ± 5.95% senescent cells) were decreased after the treatment of sEVs (63.24% ± 2.99% senescent cells), sEVs^siMDM2^ (46.86% ± 2.39% senescent cells), and WPD‐sEVs^siMDM2^ (31.13% ± 3.14% senescent cells) (Figure 3E,F). Importantly, WPD‐sEVs^siMDM2^ treatment displayed greater scavenging action on senescent cells in contrast to sEVs^siMDM2^ treatment group (*P* = 0.0445). Similarly, immunofluorescence for senescence biomarker P16^INK4a^ demonstrated WPD‐sEVs^siMDM2^ treatment (28.99% ± 4.15%) more effectively reduced the percentage of P16^INK4a^‐positive senescent chondrocytes (69.29% ± 3.72%), compared to the sEVs^siMDM2^ treatment group (47.55% ± 3.69%, *P* = 0.0022) (Figure 3G,H). *Cdkn1a* and *Cdkn2a*, which are the core senescence‐associated genes were also up‐regulated in doxorubicin‐induced chondrocytes and effectively inhibited after treatment of sEVs, sEVs^siMDM2^, or WPD‐sEVs^siMDM2^, while WPD‐sEVs^siMDM2^ showed greater inhibitory effect compared to the sEVs^siMDM2^ treatment group (Figure S8A,B, Supporting Information). Meanwhile, up‐regulation of catabolism markers (*Mmp13* and *Adamts5*) (Figure S8C,D, Supporting Information) and SASP factors (*Il6* and *Tnfα*) (Figure S8E,F, Supporting Information) in doxorubicin‐induced chondrocytes were effectively abolished after the treatment of sEVs, sEVs^siMDM2^, or WPD‐sEVs^siMDM2^, and WPD‐sEVs^siMDM2^ more effectively inhibited the production of SASP factors than sEVs^siMDM2^. Enhanced clearance effect of senescent chondrocytes was further confirmed by significantly reduced immunostaining for P21 (Figure S9, Supporting Information) and nuclear γH2AX (Figure 3G) in chondrocytes after WPD‐sEVs^siMDM2^ treatment.

To investigate the molecular mechanism of multifunctionally engineered sEVs on attenuating chondrocyte senescence, we further detected MDM2 expression in doxorubicin‐induced chondrocytes after different treatments. Exposure to doxorubicin markedly promoted the expression of MDM2 in chondrocytes, and both sEVs^siMDM2^ and WPD‐sEVs^siMDM2^ treatment inhibited this effect (Figure S9C,D, Supporting Information). Further, we found WPD‐sEVs^siMDM2^ showed a stronger inhibitory effect on MDM2 expression (Figure S9C,D, Supporting Information). Consistently, the results in PCR analysis demonstrated that both sEVs^siMDM2^ and WPD‐sEVs^siMDM2^ treatment down‐regulated the mRNA expression of *Mdm2* in doxorubicin‐induced chondrocytes, while WPD‐sEVs^siMDM2^ presented an enhanced inhibitory effect than sEVs^siMDM2^ (Figure S9E, Supporting Information). Western blot analysis results also showed WPD‐sEVs^siMDM2^ treatment effectively inhibited the protein expressions of P16^INK4a^ and P21 (Figure 3I). Additionally, we found WPD‐sEVs^siMDM2^ treatment promoted MDM2 protein expression and induced P53 phosphorylation in senescent chondrocytes (Figure 3J). To further validate the essential role of MDM2‐P53 pathway in the antiaging function of WPD‐sEVs^siMDM2^ on senescent chondrocytes, P53 phosphorylation inhibitor was applied on chondrocytes. We found the inhibitor treatment abrogated the antiaging function of WPD‐sEVs^siMDM2^ on chondrocytes as P16^INK4a^ and P21 expressions were increased (Figure 3K). As expected, P53 phosphorylation was suppressed obviously after inhibitor treatment (Figure 3L). More importantly, we found WPD‐sEVs^siMDM2^ promoted the apoptosis of senescent chondrocytes as P16^INK4a+^ and TUNEL^+^ double positive cells increased from 2.30% ± 2.37% to 31.03% ± 2.87% after WPD‐sEVs^siMDM2^ treatment (Figure S10, Supporting Information). Altogether, these results demonstrated that multifunctional modification significantly improved the antiaging effects of sEVs in vitro.

### 2.3 Full‐Thickness Human Cartilage Penetration Ability and Joint Retention Capacity of WPD‐sEVs^siMDM2^

**Figure 4:**
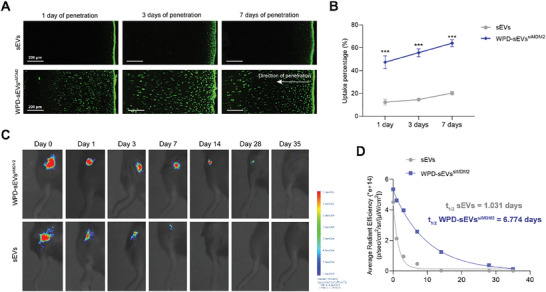
The articular cartilage penetration ability, retention capacity and cellular uptake efficiency of WPD‐sEVs^siMDM2^. A) Microscope images of DiO (Green) labeled sEVs or WPD‐sEVs^siMDM2^ across the diffusion gradient (From right to left) of normal cartilage. Scale bar: 200 µm. B) Cartilage uptake percentages of DiO‐labeled sEVs or WPD‐sEVs^siMDM2^ in cartilage explants for a 7‐day period (*n* = 3 biologically independent samples). C) Representative IVIS images of mice injected with DiR‐labeled sEVs or WPD‐sEVs^siMDM2^ over 35 days after intra‐articular injection. D) Time course of fluorescent radiant efficiency within joints in sEVs and WPD‐sEVs^siMDM2^ group. Data are fit to a one‐phase exponential decay with a common plateau based on background signal. Half‐lives were statistically different for each dataset (*P* < 0.0001) by extra sum of squares *F*‐test.

Given the abundant negatively charged biomacromolecules in cartilage matrix, human articular cartilage explants (Φ 5 × 1 mm) were used to examine whether WPD modification could enhance the penetration ability of sEVs. Cartilage explants were stuck in a customized chamber using the one‐way transport penetration mould, and 200 µl DiO labeled unmodified sEVs (1 × 10^10^ particles ml^−1^) or WPD‐sEVs^siMDM2^ suspensions (1 × 10^10^ particles ml^−1^) were added to the left side of penetration mould while 200 µl PBS were added to the right side. After penetration ex vivo for different times including 1, 3, and 7 days, the cartilage explants were cryo‐sectioned and observed by laser confocal microscopy. As shown in **Figure** **4**A, unmodified sEVs still concentrated in the superficial zone of the cartilage explants after penetration for 3 days, indicating a very limited penetration ability. In contrast, WPD‐sEVs^siMDM2^ had diffused into the deep zone of cartilage explants after 1‐day penetration (Figure 4A). Significantly, WPD‐sEVs^siMDM2^ had distributed throughout the cartilage explants after penetration for 3 days (Figure 4A). For the uptake evaluation in cartilage explants, DiO labeled unmodified sEVs (1 × 10^10^ particles ml^−1^) or WPD‐sEVs^siMDM2^ (1 × 10^10^ particles ml^−1^) were incubated with these cartilage explants (Φ 5 × 1 mm), and the cartilage uptake efficiency was analyzed as the percentage of DiO‐labeled sEVs or WPD‐sEVs^siMDM2^ from 200 µl total solution to the cartilage explants. The results in Figure 4B and Table S2 (Supporting Information) demonstrated that WPD‐sEVs^siMDM2^ exhibited significantly enhanced cartilage uptake ratio than unmodified sEVs at all time points, which was in consistent with the one‐way penetration assay. In particular, after incubation for 24 h, 47.33% ± 4.50% WPD‐sEVs^siMDM2^ were taken up in cartilage explants while it was only 12.33% ± 2.05% unmodified sEVs were taken up. After 7 days penetration, 64.00% ± 2.45% WPD‐sEVs^siMDM2^ were taken up and 20.33% ± 1.25% sEVs were taken up in cartilage explants. These results convincingly verified that WPD‐sEVs^siMDM2^ are capable of penetrating into the deep zone of the articular cartilage and exhibit improved cartilage penetration ability.

Next, we directly injected unmodified sEVs (1 × 10^10^ particles ml^−1^) or WPD‐sEVs^siMDM2^ (1 × 10^10^ particles ml^−1^) labeled with a near‐infrared fluorescence dye DiR into the knee joint to assess the retention capacity. In vivo imaging analysis was used to further evaluate the joint retention time of WPD‐sEVs^siMDM2^. For a 35‐day period, DiR fluorescence signal in the mouse knee joint was serially detected and analyzed. Immediately after intra‐articular injection, we detected bright fluorescence signals in both sEVs group and WPD‐sEVs^siMDM2^ group (Figure 4C). However, the DiR fluorescence signal was dropped dramatically 1 day after injection in sEVs group, while WPD‐sEVs^siMDM2^ group showed significant slower reduction in fluorescence intensity during the same period, indicating that most of the unmodified sEVs were quickly cleared from the joint by the fast clearance system. Notably, the fluorescence signal was still detectable in WPD‐sEVs^siMDM2^ group at day 28 after intra‐articular injection, whereas the fluorescence signal was barely detectable in unmodified sEVs group at day14 after injection, suggesting that the retention time of WPD‐sEVs^siMDM2^ in knee joint is much longer than that of unmodified sEVs. Applying the fluorescence signal data to a single‐phase exponential decay function, we found that the unmodified sEVs had a joint half‐life of 1.031 days, whereas WPD‐sEVs^siMDM2^ significantly prolonged the half‐life to 6.774 days (Figure 4D). Overall, these results demonstrated that multifunctional modification significantly extended the joint retention ability and enhanced the cartilage penetration capacity of sEVs.

### 2.4 Efficacy of WPD‐sEVs^siMDM2^ on Chondrocyte Senescence and Cartilage Matrix Metabolic Homeostasis in PTOA Mice

**Figure 5:**
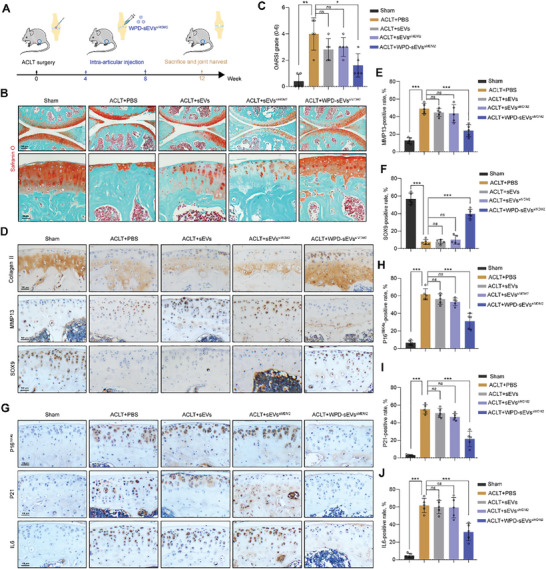
Evaluation of WPD‐sEVs^siMDM2^ for cartilage degeneration and senescent phenotype in ACLT‐induced post‐traumatic OA mouse. A) Schematic diagram depicting the experimental timeline of the intra‐articular delivery of WPD‐sEVs^siMDM2^ that elicits inflammatory responses, treatment with WPD‐sEVs^siMDM2^ (1 × 10^10^ particles ml^−1^), and subsequent analysis to evaluate cartilage degeneration and senescent phenotype. B) Representative images of Safranin O staining of articular cartilage in each group. Scale bar: 150 µm. C) Statistic data of OARSI grade in each group (*n* = 5). D) Representative images of immunohistochemical staining for matrix anabolism biomarker collagen II, catabolism marker MMP13, and chondrogenic marker SOX9 in articular cartilage. Scale bar: 100 µm. E,F) Quantitative analysis of immunohistochemical staining of MMP13 and SOX9 (*n* = 5). G) Representative images of immunohistochemical staining for senescent biomarkers (P16^INK4a^ and P21), and SASP factor IL6 in each group. Scale bar: 100 µm. H–J) Quantitative analysis of immunohistochemical staining of P16^INK4a^, P21, and IL6 (*n* = 5). Data are represented as mean ± SD. *P < 0.05, **P < 0.01, and ***P < 0.001, *ns*, not significant.

We next explored the efficacy of WPD‐sEVs^siMDM2^ for treating anterior cruciate ligament transection (ACLT)‐induced PTOA mice. In view of the significantly extended half‐life of WPD‐sEVs^siMDM2^ (from 1.031 to 6.774 days), intra‐articular injection of different treatments was performed once a month over two months starting on day 28 after ACLT surgery (**Figure** **5**A). Safranin O staining was carried out to evaluate the cartilage destruction. As shown in Figure 5B, obvious cartilage abrasion and loss was presented in PBS‐treated PTOA mice, and these features were also found in sEVs‐treated (1 × 10^10^ particles ml^−1^) and sEVs^siMDM2^‐treated (1 × 10^10^ particles ml^−1^) PTOA mice. In contrast, WPD‐sEVs^siMDM2^ treatment (1 × 10^10^ particles ml^−1^) effectively slowed the cartilage degeneration and maintained cartilage matrix metabolic homeostasis in PTOA mice. The Osteoarthritis Research Society International (OARSI) scoring system was also applied to quantify the OA severity. Our results showed there was no significant difference between PBS‐treated group (4.00 ± 1.10), sEVs‐treated group (2.80 ± 0.75, *P* = 0.18), and sEVs^siMDM2^‐treated group (3.00 ± 0.63, *P* = 0.17) (Figure 5C). However, the OARSI score of WPD‐sEVs^siMDM2^‐treated group (1.60 ± 0.80) was significantly lower than that of PBS‐treated group (*P* = 0.02) (Figure 5C), validating the cartilage protective effects of WPD‐sEVs^siMDM2^ in PTOA mice. Subsequently, immunohistochemical staining for collagen II, MMP13, and SOX9 was performed to evaluate the regulative effect of WPD‐sEVs^siMDM2^ on cartilage anabolism and catabolism. We found the expressions of collagen II and SOX9 was increased and MMP13 expression was decreased in ACLT‐induced PTOA mice after the treatment of WPD‐sEVs^siMDM2^, while sEVs and sEVs^siMDM2^ treatment showed no significant difference to PBS‐treated group (Figure 5D‐F).

Moreover, the antiaging effects of WPD‐sEVs^siMDM2^ in articular cartilage of ACLT‐induced PTOA mice were evaluated. Consistent with our in vitro results, immunohistochemical staining for P16^INK4a^ showed that the percentage of senescent cells was increased after ACLT surgery (from 6.62% ± 2.53% to 61.76% ± 5.81%), while only WPD‐sEVs^siMDM2^ treatment effectively decreased the number of senescent cells (31.12% ± 7.92%) (Figure 5G,H). Next, enhanced clearance function of senescent cells was further confirmed by the reduced expressions of P21 (Figure 5G,I), SASP factors (IL6 and TNFα) (Figure 5G,J, Figure S11A,B, Supporting Information), and HMGB1 (Figure S11A,C, Supporting Information) in WPD‐sEVs^siMDM2^‐treated PTOA mice, compared to the sEVs^siMDM2^ treatment group. To confirm the role of MDM2‐P53 pathway in the antiaging function of WPD‐sEVs^siMDM2^ in OA cartilage in vivo. The expression of MDM2 in articular cartilage was further evaluated in PTOA mice after different treatments. immunohistochemical staining results showed MDM2‐positive chondrocytes was increased in PBS‐treated PTOA mice (from 2.55% ± 1.60% to 57.56% ± 8.09%), while only WPD‐sEVs^siMDM2^ treatment effectively down‐regulated the percentage of MDM2‐positive chondrocytes (19.64% ± 3.18%) (Figure S11D,E, Supporting Information). Additionally, we found only WPD‐sEVs^siMDM2^ treatment effectively inhibited the expression of P53 and promoted the expression of p‐P53 in mice cartilage (Figure S11F,G, Supporting Information).

Interestingly, our results also demonstrated WPD‐sEVs^siMDM2^ treatment markedly decreased the number of inflammatory cells in the synovium (Figure S12A, Supporting Information) and synovial inflammation score (from 1.80 ± 0.40 to 0.60 ± 0.49, *P* = 0.03) (Figure S12B Supporting Information) of ACLT‐induced PTOA mice, suggesting therapeutic effects on articular cartilage mediated by WPD‐sEVs^siMDM2^ could ameliorate synovitis in the joint. In addition, our results showed WPD‐sEVs^siMDM2^ treatment decreased subchondral bone plate score (*P* = 0.0476) and bone volume score (*P* = 0.0397) in PTOA mice knee joint (Figure S12C‐E, Supporting Information). Together, these in vivo results suggested that WPD‐sEVs^siMDM2^ treatment can alleviate senescent phenotype and maintain matrix metabolic homeostasis in ACLT‐induced PTOA mice.

### 2.5 Therapeutic Effects of WPD‐sEVs^siMDM2^ on Cartilage Senescence and Matrix Metabolism in Naturally Aged OA Mice

**Figure 6:**
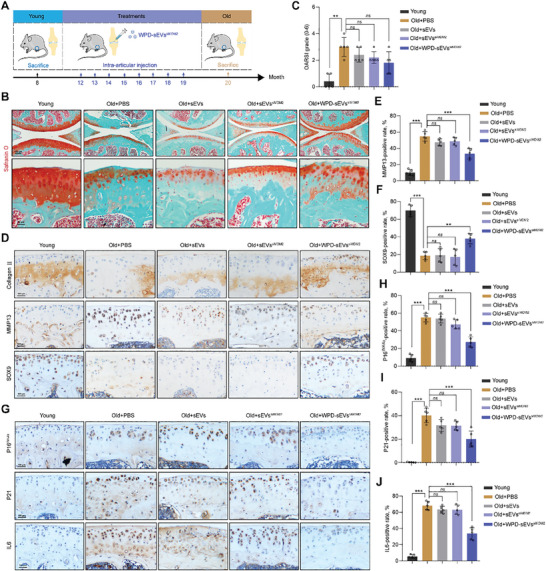
Evaluation of WPD‐sEVs^siMDM2^ for cartilage degeneration and senescent phenotype in naturally aged OA mouse model. A) Schematic illustration of processing timeline and procedures. Eight‐month‐old mice were used as the young control group and 20‐month‐old mice were used as the old control group. B) Representative images of Safranin O staining in each group. Scale bar: 150 µm. C) Statistic data of OARSI grade in each group (*n* = 5). D) Representative images of immunohistochemical staining for anabolism biomarker collagen II, catabolism marker MMP13, and chondrogenic marker SOX9 in articular cartilage of naturally aged mice. Scale bar: 100 µm. E,F) Quantitative analysis of immunohistochemical staining of MMP13 and SOX9 (*n* = 5). G) Representative images of immunohistochemical staining for senescent biomarkers (P16^INK4a^ and P21), and SASP factor IL6 in each group. Scale bar: 100 µm. H–J) Quantitative analysis of immunohistochemical staining of P16^INK4a^, P21, and IL6 (*n* = 5). Data are represented as mean ± SD. **P < 0.01 and ***P < 0.001, *ns*, not significant.

Aging in mice causes the development of spontaneous OA, similar to what occurs in human patients. We therefore investigated the antiaging effects of WPD‐sEVs^siMDM2^ treatment in naturally aged mice. Starting at the age of 12‐month‐old, WPD‐sEVs^siMDM2^ (1 × 10^10^ particles ml^−1^) was intra‐articularly injected into the joint cavity of mice once a month for 8 months (**Figure** **6**A). Safranin O staining results showed obvious cartilage destruction was observed in PBS‐treated 20‐month‐old naturally aged mice, and only WPD‐sEVs^siMDM2^ treatment significantly reduced cartilage loss and maintained the intact structure of articular cartilage in aged mice (Figure 6B). In aged mice, WPD‐sEVs^siMDM2^ treatment obviously reduced OARSI score (from 3.00 ± 0.63 to 1.80 ± 0.75), but it showed no significant difference (*P* = 0.1032) (Figure 6C). Additionally, the expression of anabolism‐related marker collagen II and SOX9 was significantly increased and catabolism‐related marker MMP13 was reduced in aged mice after WPD‐sEVs^siMDM2^ treatment (Figure 6D‐F), suggesting a certain degree of enhanced cartilage protective effects of multifunctionally engineered sEVs.

Similar to the antiaging effects of WPD‐sEVs^siMDM2^ in ACLT‐induced PTOA mice, only WPD‐sEVs^siMDM2^ treatment effectively eliminated P16^INK4a^‐positive senescent cells (from 55.04% ± 4.31% to 27.28% ± 5.30%) in articular cartilage of naturally aged mice (Figure 6G,H). Furthermore, the expressions of P21 (Figure 6G,I), SASP factors (IL6 and TNFα) (Figure 6G,J, Figure S13A,B, Supporting Information), and HMGB1 (Figure S13A,C, Supporting Information) were notably increased in PBS‐treated aged mice, while only WPD‐sEVs^siMDM2^ treatment significantly reduced the expressions of P21, HMGB1, and SASP factors. To further verify the role of MDM2‐P53 pathway in naturally aged mice, the expression of MDM2 in articular cartilage of aged mice was analyzed by immunohistochemical staining after different treatments. In particular, the percentage of MDM2‐positive chondrocytes in articular cartilage was significantly up‐regulated in PBS‐treated aged mice (65.71% ± 5.61%), compared to the young mice (4.84% ± 1.36%). Consistent with our results in ACLT‐induced PTOA mice, only WPD‐sEVs^siMDM2^ treatment effectively decreased the percentage of MDM2‐positive chondrocytes in aged mice (from 65.71% ± 5.61% to 33.52% ± 5.45%) (Figure S13D,E, Supporting Information). Additionally, our results showed WPD‐sEVs^siMDM2^ treatment effectively inhibited the expression of P53 and up‐regulated the expression of p‐P53 in mice cartilage (Figure S13F,G, Supporting Information).

In consistent with the results in ACLT‐induced PTOA mice, Hematoxylin & Eosin (H&E) staining of synovium results demonstrated only WPD‐sEVs^siMDM2^ treatment decreased the synovial inflammation score of aged mice (from 1.60 ± 0.49 to 0.60 ± 0.49, *P* = 0.0794) (Figure S14A,B, Supporting Information). Moreover, we found WPD‐sEVs^siMDM2^ treatment decreased subchondral bone plate score and bone volume score in naturally aged mice, but it also presented no obvious difference compared with the PBS‐treated mice (Figure S14C–E, Supporting Information). Altogether, our data indicated that WPD‐sEVs^siMDM2^ treatment effectively alleviated senescent phenotype and inhibited cartilage matrix catabolism in naturally aged mice.

### 2.6 Efficacy of WPD‐sEVs^siMDM2^ on Senescent Phenotype and Matrix Anabolism in Human OA Cartilage Explants

**Figure 7:**
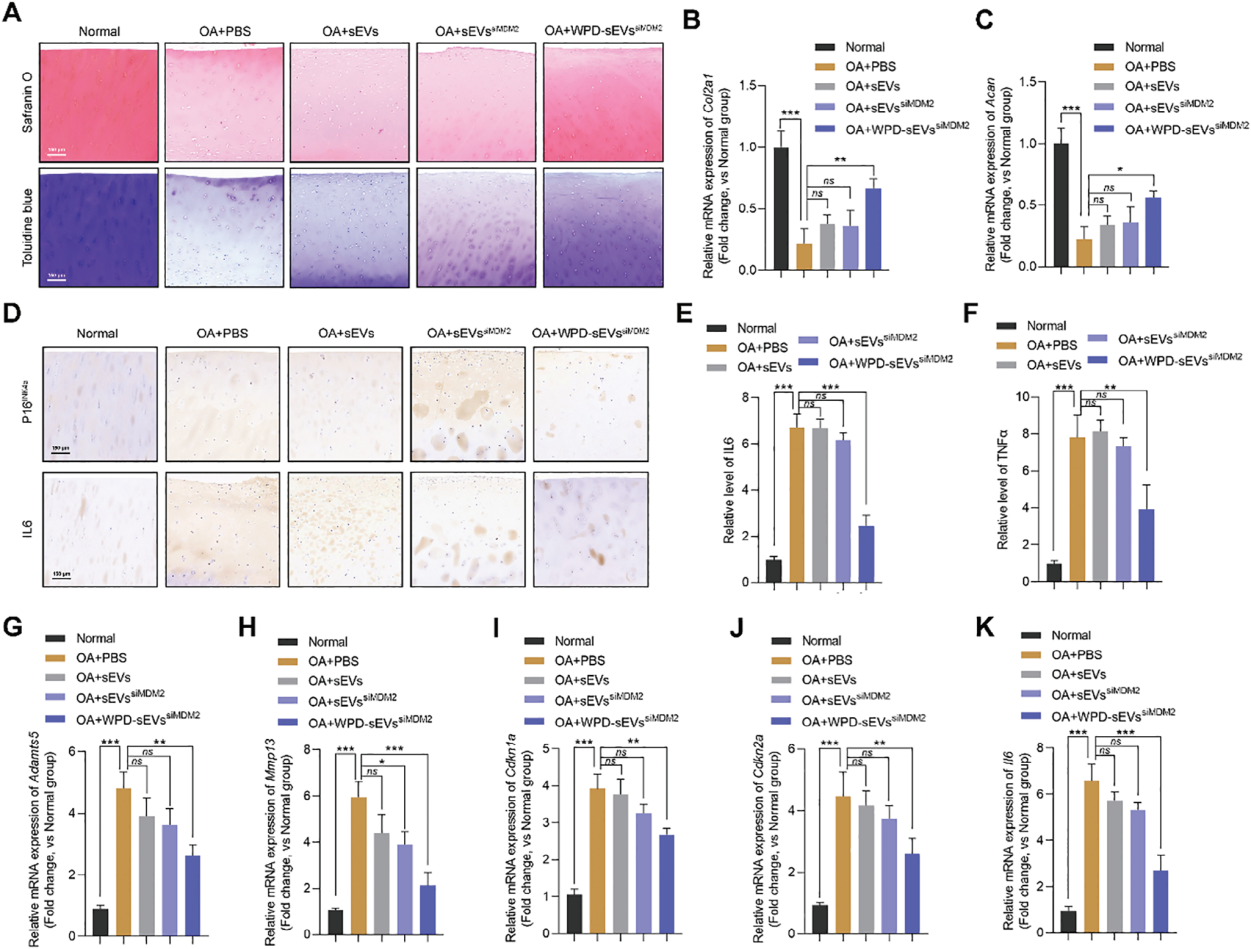
Effect of WPD‐sEVs^siMDM2^ on cartilage destruction and senescent phenotype in human OA cartilage explants. A) Representative micrographs of Safranin O and Toluidine blue staining after 7 days treatment with WPD‐sEVs^siMDM2^. Scale bar, 50 µm. B,C) Relative mRNA expressions of extracellular matrix anabolism markers (*Col2a1* and *Acan*) after treatment of WPD‐sEVs^siMDM2^ were determined by PCR analysis (*n* = 3). D) Representative images of immunohistochemical staining for P16^INK4a^ and IL6 in cartilage explants. E,F) ELISA analysis for SASP factors (IL6 and TNFα) in cartilage explants. G,H) Relative mRNA expressions of catabolism‐related markers *Adamts5* and *Mmp13* after treatment of WPD‐sEVs^siMDM2^ were determined by PCR analysis (*n* = 3). I,J) Relative mRNA expressions of aging‐related markers *Cdkn2a* and *Cdkn1a* after treatment of WPD‐sEVs^siMDM2^ were determined by PCR analysis (*n* = 3). K) Relative mRNA expression of SASP factor *Il6* after treatment of WPD‐sEVs^siMDM2^ was detected by PCR analysis (*n* = 3). Data are represented as mean ± SD. *P < 0.05, **P < 0.01, and ***P < 0.001, *ns*, not significant.

Last, to validate the feasibility of the versatile engineered sEVs strategy in clinical OA patients, we evaluated the effects of WPD‐sEVs^siMDM2^ in a three‐dimensional environment by using ex vivo cultured OA articular cartilage explants from patients undergoing total knee arthroplasty, and OA‐affected cartilage explants were divided into four groups that received PBS, sEVs (1 × 10^10^ particles ml^−1^), sEVs^siMDM2^ (1 × 10^10^ particles ml^−1^), and WPD‐sEVs^siMDM2^ (1 × 10^10^ particles ml^−1^) treatments, respectively. Non‐OA affected normal cartilage was used as the control group. As shown in **Figure** **7**A, significant proteoglycan loss and cartilage degradation was observed in OA‐affected cartilage in contrast to the non‐OA affected cartilage, while multifunctionally modification of sEVs effectively promoted the production of cartilage matrix and ameliorated cartilage destruction (Figure 7A), further confirming the enhanced protective effects of WPD‐sEVs^siMDM2^ treatment. Consistently, the mRNA expressions of *Acan* and *Col2a1* were markedly down‐regulated in OA‐affected cartilage, while only WPD‐sEVs^siMDM2^ treatment effectively increased the mRNA expressions of *Col2a1* (Figure 7B) and *Acan* (Figure 7C). Consistent with our in vivo and in vitro results, immunohistochemical staining for P16^INK4a^ and IL6 showed WPD‐sEVs^siMDM2^ treatment effectively decreased the expressions of P16^INK4a^ and IL6 in OA cartilage (Figure 7D). Enzyme‐Linked Immunosorbent Assay (ELISA) results further confirmed that the protein expressions of SASP factors (IL6 and TNFα) were significantly down‐regulated after the treatment of WPD‐sEVs^siMDM2^ (Figure 7E,F). Next, senescent phenotype was also confirmed in OA‐affected cartilage by the enhanced gene expressions of catabolism‐related genes (*Adamts5* and *Mmp13*) (Figure 7G,H), senescence marker (*Cdkn1a* and *Cdkn2a*) (Figure 7I,J), and pro‐inflammatory factor *Il6* (Figure 7K). In contrast to sEVs‐treated group and sEVs^siMDM2^‐treated group, WPD‐sEVs^siMDM2^ treatment more effectively suppressed the mRNA expressions of these senescence‐related genes in OA‐affected cartilage, corroborating our results from cultured human chondrocytes, PTOA mice, and naturally aged mice. Together, these data provide ex vivo evidence that WPD‐sEVs^siMDM2^ treatment has significant antiaging effects and protective action on senescent chondrocytes and OA cartilage.

## 3 Discussion

In the present study, we constructed a novel multifunctionally engineered MSC‐sEVs for OA treatment. First, induced pluripotent stem cells derived MSCs were used to harvest sEVs as these MSCs are easily to be produced in large scale to generate sufficient sEVs for application and avoid the problem of cell source variation. Next, MSC‐sEVs were loaded with siMDM2 to enhance its antiaging function and further modified with a cationic cartilage‐targeting peptide WPD to improve its cartilage‐targeting ability. We systematically evaluated the feasibility, safety, and efficacy of WPD‐sEVs^siMDM2^ in OA treatment. Our results verified multifunctional modification showed no impact on the morphology and contents of MSC‐sEVs, and WPD‐sEVs^siMDM2^ presented a good stability in PBS and HA or CS solutions, indicating an excellent biocompatibility and can be used as a safe modification strategy for OA treatment. Furthermore, multifunctional modification improved the cellular uptake capacity of MSC‐sEVs in chondrocytes, and thus enhanced the antiaging effects. Impressively, versatile modification significantly enhanced the cartilage penetration ability and extended the joint retention time of MSC‐sEVs in vivo. In both ACLT‐induced PTOA mice and naturally aged mice, we further confirmed multifunctionally modified MSC‐sEVs more effectively eliminated senescent cells, inhibited SASP factors, maintained matrix metabolic homeostasis, and retarded OA progression, compared to the unmodified MSC‐sEVs. Additionally, WPD‐sEVs^siMDM2^ successfully reversed senescent phenotype and promoted cartilage matrix anabolism in ex vivo cultured human OA cartilage explants. Overall, our findings suggest that WPD‐sEVs^siMDM2^ constitute an effective versatile antiaging nanoplatform for OA treatment.

To the best of our knowledge, this is the first study describing the application of MSC‐sEVs loaded with antiaging cargo and engineered with a cartilage‐targeting peptide to substantially enhance the antiaging effects of MSC‐sEVs by targetedly eliminating senescent chondrocytes and reversing senescent phenotype in OA cartilage. In recent years, sEVs derived from different tissues‐sourced MSCs (bone marrow, adipose tissue, synovium, and infrapatellar fat pad) have been postulated as a promising strategy for cartilage regeneration and OA treatment.^[^ ^16^ ^]^ Numerous studies have reported that MSC‐sEVs are encapsulated with abundant bioactive molecules, including anti‐inflammatory cytokines, growth factors, and chondro‐protective factors.^[^ ^17^ ^]^ Thus, MSC‐sEVs have been extensively explored for the treatment of OA due to their ability to alleviate inflammatory responses and promote cartilage matrix anabolism. However, persistent production and secretion of SASP factors from accumulated senescent chondrocytes in OA cartilage can still create vicious microenvironment in the joint and abrogate the protective effects of MSC‐sEVs. Unlike previously reported MSC‐sEVs‐based strategies for OA treatment, WPD‐sEVs^siMDM2^ can targetedly deliver siMDM2 to chondrocytes, which can effectively eliminate senescent cells, reverse senescent phenotype, and maintain matrix metabolic homeostasis in OA cartilage.

Chondrocyte senescence is associated with the progression of post‐traumatic and aging‐related OA, positioning senescent chondrocytes as key targets for OA treatment.^[^ ^18^ ^]^ In previous studies, ACLT‐ and destabilization of the medial meniscus (DMM)‐induced PTOA animal models were usually used to evaluate the chondro‐protective effects of therapeutics.^[^ ^19^ ^]^ In our study, both ACLT‐induced PTOA mice and naturally aged mice were used to confirm the antiaging function of WPD‐sEVs^siMDM2^, and WPD‐sEVs^siMDM2^ were applied after the occurrence of senescent phenotype in articular cartilage. Our results verified WPD‐sEVs^siMDM2^ successfully reduced the percentage of senescent chondrocytes, reversed senescent phenotype, and retarded cartilage degeneration in both PTOA mice and naturally aged mice. Besides, rodents such as mice and rats were usually used to establish OA models for pre‐clinical evaluation of therapeutics, while these animals possess a relatively thinner thickness of articular cartilage (∼100 µm). However, human articular cartilage shows much larger thickness (∼2.35 mm) than that of rodents, which may further limit the efficacy of drugs and hinder the clinical translation. In this work, articular cartilage from OA patients undergoing knee replacement surgery were harvested and cultured ex vivo. We confirmed that WPD‐sEVs^siMDM2^ effectively reversed senescent phenotype and maintained matrix metabolic homeostasis in cultured OA cartilage explants, indicating a potential for clinical translation.

Gene editing and small molecule drugs intervention are effective strategies for senescent chondrocytes regulation. According to published studies,^[^ ^1^, ^19^, ^20^ ^]^ MDM2 inhibition is a potential therapeutic candidate for degenerative diseases through promoting the apoptosis of senescent cells. Previous research has shown that elimination of senescent cells by MDM2 specific inhibitor UBX0101 effectively inhibited SASP factors production and promoted cartilage regeneration in PTOA mice.^[^ ^19^, ^20^ ^]^ However, UBX0101 failed in the clinical trial for OA treatment probably owing to low bioavailability, off‐target effects, and instability in the joint. Thus, a reasonable and efficient senescent chondrocytes clearance method is a promising strategy for OA treatment by creating pro‐chondrogenic microenvironment in the joint and alleviating cartilage degeneration. RNAi technology provides a unique opportunity to specifically inhibit targeted gene with reduced off‐target effect and improved therapeutic function.^[^ ^21^ ^]^ The clinical application of siRNA‐related medicine is strongly supported by recent success of clinical trials and US Food and Drug Agency approval of several drugs. Nevertheless, direct intra‐articular injection of siRNA in animals was not durable because of the multiple barriers in the joint.^[^ ^22^ ^]^ Hence, a long‐lasting, joint‐localized, and chondrocyte‐targeting strategy based on RNAi technology is highly valuable for OA therapy. Following the discovery of their role in the communication between different tissues and cells by delivering functional cargos, natural sEVs have been regarded as promising nanocarriers for developing novel therapeutic delivery platforms which can address the drawbacks of synthetic nanocarriers including nanoparticles, liposomes, quantum dots. Compared to the synthetic nanocarriers, sEVs are advantageous because of the low cytotoxicity and immunogenicity, natural affinity, and high tissue permeability.^[^ ^6a^ ^]^ In addition, lipids and proteins on sEVs surface can be easily engineered for greater therapeutic function. In the present study, siMDM2 was encapsulated into MSC‐sEVs and then engineered with cationic cartilage‐targeting peptide WPD to construct a multifunctionally modified WPD‐sEVs^siMDM2^. Notably, we were excited to confirm that MSC‐sEVs‐mediated encapsulation protected siMDM2 from degradation, consistent with previous studies that the membrane structure of sEVs can maintain the stability of cargos in vivo.^[^ ^23^ ^]^ Versatile modification significantly enhanced the antiaging effects of MSC‐sEVs in vitro and in vivo by eliminating senescent chondrocytes and inhibiting SASP factors secretion. As an extracellular alarmin whose nuclear expression precedes the secretion of SASP components in cells undergoing senescence, we found HMGB1 was inhibited in OA mice cartilage and WPD‐sEVs^siMDM2^ treatment effectively increased HMGB1 expression. However, some other studies showed HMGB1 expression is up‐regulated in OA cartilage,^[^ ^24^ ^]^ which is inconsistent with our results. Further research is needed to explore the function HMGB1 in chondrocyte senescence and OA progression. Additionally, most studies reported that the function of sEVs loaded with siRNA has mainly depended on the siRNA cargo whereas the sEVs only acted as the nanocarrier. In contrast, the WPD‐sEVs^siMDM2^ took advantage of the intrinsic anti‐inflammatory effects of MSC‐sEVs and collaborated with the loaded siMDM2 to notably enhance the antiaging efficacy, which can concurrently eliminate senescent chondrocytes and inhibit SASP factors production. Such comprehensive studies will shed light on developing disease‐modifying drugs that benefit OA patients and other degenerative diseases.

Intra‐articular delivery of therapeutics has been termed a major challenge owing to the rapid clearance of the drugs by the fast clearance system and thus leading to the short residence time in the joint cavity, accounting for low bioavailability. Thus, repeated intra‐articular injection were required to ensure the efficacy of drugs for OA treatment, while frequent intra‐articular injection may cause joint infection, soft tissue injury, and systematic adverse effects. Additionally, given the dense collagen II network with a pore size of about 60 nm in the superficial zone,^[^ ^13^ ^]^ while most of the chondrocytes reside in the middle and deep zone of articular cartilage, further leading to the failure of therapeutics. Further, taking into consideration the complex structure of the joint, sEVs‐based delivery nanoplatform offers a promising cell‐free therapy, with a highly customizable system that can be engineered to maximize targeted delivery of antiaging bioactive molecules for OA cartilage. Recently, a bone‐targeting peptide DSPE‐PEG‐Mal‐Cys‐SDSSD was applied to modify MSC‐sEVs and then loaded with siRNA Shn3 to construct a sEVs‐based delivery system.^[^ ^23b^ ^]^ This bone‐targeting delivery system obviously enhanced osteogenic differentiation, inhibited osteoclast formation, and serve as a promising therapy for osteoporosis treatment. Xia et al. reported chondrocyte affinity peptide‐modified sEVs can efficiently deliver miR‐140 to deep zone of articular cartilage, inhibit cartilage matrix catabolism, and thus alleviate rat OA development.^[^ ^10c^ ^]^ Inspired by these studies, we synthesized a novel cationic collagen II‐targeting peptide WPD and further engineered sEVs^siMDM2^ for developing a versatile antiaging nanoplatform with senescent chondrocytes‐targeted clearance ability. Compared with other cartilage‐targeting peptide, WPD modified sEVs not only showed cartilage‐targeting function but also presented positive surface charge (9.13 ± 1.31 mV). Our results validated WPD modification substantially improved the cartilage penetration ability and extended the joint retention time of MSC‐sEVs, suggesting a notably enhanced bioavailability in the joint. Because of this, we further confirmed that WPD‐sEVs^siMDM2^ more effectively reduced the percentage of senescent chondrocytes and alleviated cartilage degradation in ACLT‐induced PTOA mice and naturally aged mice, compared to unmodified sEVs and sEVs^siMDM2^. Our results highlight that versatile modification notably enhanced the antiaging effect of MSC‐sEVs by targetedly delivering siMDM2 and other internal bioactive factors from MSC‐sEVs to articular chondrocytes. Although our results provide robust pre‐clinical data supporting the therapeutic efficacy and safety of multifunctionally engineered MSC‐sEVs by using two OA mice models and ex vivo cultured cartilage explants, additional studies are needed to evaluate efficacy and safety in larger OA animal models before this strategy can be translated into the clinic.

## 4 Conclusion

**Figure 8:**
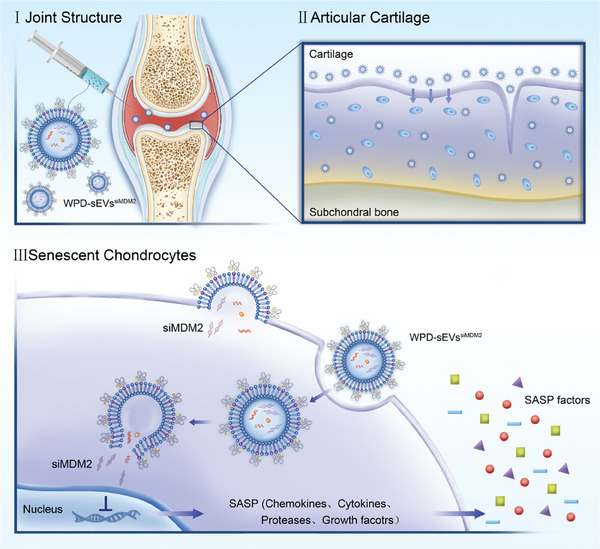
Schematic of multifunctionally engineered WPD‐sEVs^siMDM2^ for the treatment of OA. The WPD‐sEVs^siMDM2^ targeted the articular cartilage in OA joints after intra‐articular injection and efficiently penetrated into the deep zone of cartilage. Subsequently, WPD‐sEVs^siMDM2^ reversed senescent phenotype by delivering siMDM2 and other bioactive molecules in original MSC‐sEVs. Specifically, WPD‐sEVs^siMDM2^ effectively eliminated senescent chondrocytes and inhibited SASP factors secretion, thus maintaining cartilage matrix metabolism homeostasis and alleviating OA progression.

In this study, we describe an effective strategy to generate a multifunctionally engineered MSC‐sEVs‐based cell‐free therapy for OA treatment through targetedly eliminating senescent chondrocytes and maintaining cartilage matrix metabolic homeostasis (**Figure** **8**). Taking advantage of the native anti‐inflammatory effects of MSC‐sEVs, siMDM2 and cartilage‐targeting peptide WPD were further applied to modify MSC‐sEVs for orchestrating a comprehensive antiaging strategy for OA treatment. The multifunctional modification can drive the natural MSC‐sEVs to rapidly adsorb on the articular cartilage surface and pass through the negatively charged dense matrix efficiently, then targetedly delivering siMDM2 to senescent cells for effective clearance. In particular, the versatile engineered MSC‐sEVs exhibited enhanced articular cartilage penetration, improved joint retention capacity, and notable antiaging function. Importantly, WPD‐sEVs^siMDM2^ treatment efficiently reduced the percentage of senescent chondrocytes, inhibited the secretion of SASP factors, and maintained cartilage metabolic homeostasis in vitro and in vivo. Moreover, we confirmed the antiaging function of WPD‐sEVs^siMDM2^ on chondrocytes was closely associated with the MDM2‐P53 pathway‐mediated apoptosis inducing effect. From a future clinical perspective, we further confirmed WPD‐sEVs^siMDM2^ treatment successfully reversed senescent phenotype and promoted cartilage matrix anabolism in ex vivo cultured human OA cartilage explants. These findings clearly show that targeted elimination of senescent chondrocytes can be a promising strategy to delay OA progression and that the newly developed WPD‐sEVs^siMDM2^ holds great potential for OA treatment in clinical practice. In addition, MSC‐sEVs can be modified by different targeting ligands and diverse drugs for the treatment of other age‐associated diseases, which will provide a new drug delivery strategy for precision medicine.

## 5 Experimental Section

### Reagents

WPD and rhodamine B labeled WPD were purchased from ChinaPeptides (Suzhou, China). siMDM2 was designed and synthesized by Hanbio (Shanghai, China). Doxorubicin, bovine serum albumin (BSA), and dimethyl sulfoxide (DMSO) were obtained from Sigma‐Aldrich (MO, USA). The serum‐free ncMission hMSC Medium was obtained from Shownin Biotechnologies Co., Ltd. (RP02010, China). Dulbecco's modified Eagle's medium (DMEM)‐F12 and PBS were obtained from HyClone (UT, USA). Fetal bovine serum (FBS), collagenase II, and Penicillin–Streptomycin were purchased from Gibco (CA, USA). Lipid membrane fluorescent dyes DiO and DiR were purchased from Thermo Fisher Scientific (MA, USA). 4′,6‐Diamidino‐2‐phenylindole (DAPI) was purchased from Beyotime Biotechnology (Jiangsu, China). P53 phosphorylation inhibitor 17‐DMAG was obtained from MedChemEexpress (HY‐10389, China). The information of primary and secondary antibodies for Western blot analysis, immunofluorescent staining, and immunohistochemical staining was listed in Table S3 (Supporting Information).

### Isolation and Characterization of Versatile Engineered MSC‐sEVs

sEVs derived from induced pluripotent stem cells derived MSCs were used in this study. The iPSC cell line (iPS‐S‐01) was provided by the Institute of Biochemistry and Cell Biology of the Chinese Academy of Sciences. Briefly, the medium was replaced with a serum‐free MSC culture medium containing basal medium (Nuwacel Nova Missoin Basal Medium, Nuwacell Biotechnology, RP02010‐01) and supplement (Nuwacell Nova Missoin Supplement, Nuwacell Biotechnology, RP02010‐02) after 14 days of culturing in iPSCs culture medium (Nuwacell Biotechnology, RP01001). Then, the surface antigens of MSCs were analyzed by flow cytometry. The cells were continually passaged after reaching 80% confluence, and cells from passages five to ten were used in the subsequent experiments. After reaching 80% confluency, MSC‐sEVs were obtained by the serial ultracentrifugation method.^[^ ^15^, ^25^ ^]^ Briefly, the culture medium was centrifuged at 300×*g* for 10 min and 2000×*g* for 30 min under 4 °C. Then large EVs were removed by centrifugated at 10 000×*g* for 60 min and filtration through a 0.22 µm filter (Millipore, USA). Next, the supernatant was ultracentrifuged at 100 000×*g* for 70 min under 4 °C (ultracentrifuge Optima XPN with a SW32 Ti rotor, Beckmann, USA). After removing the supernatant, sEVs pellets were resuspended in PBS and further ultracentrifuged at 100 000×*g* for 70 min under 4 °C to harvest the pelleted sEVs.

To construct multifunctionally engineered MSC‐sEVs, siMDM2 (Table S4, Supporting Information) was loaded into MSC‐sEVs by the classical electroporation method.^[^ ^10^, ^26^ ^]^ Briefly, 10 µl MSC‐sEVs (1 × 10^12^ particles ml^−1^) and 100 µg siMDM2 were lightly mixed in 400 µl of electroporation buffer (1.15 mM potassium phosphate, pH = 7.2, 21% Optiprep, 25 mM potassium chloride) and electroporated in a 4 mm cuvette by the electroporation apparatus with a 0.35‐s pulse 20 times at 700 V as previously described.^[^ ^23b^ ^]^ Then, the mixture was incubated for 30 min at 37 °C to recover the membrane structure of MSC‐sEVs. Finally, free siMDM2 was removed from the mixture by ultracentrifugation for 70 min at 100 000×*g* under 4 °C, and the sEVs^siMDM2^ pellets were resuspended in PBS.

To achieve cartilage‐targeting ability and surface charge reverse, sEVs^siMDM2^ (1 × 10^11^ particles ml^−1^) were then incubated with the PBS solution containing various concentration of WPD (or rhodamine B labeled WPD) for 60 min at room temperature.^[^ ^15^, ^27^ ^]^ After incubation, 1 ml mixed solution were diluted with 100 ml sterile PBS to eliminate WPD aggregation. Then, the diluted solutions were purified by ultracentrifugation at 100 000×*g* for 70 min to obtain WPD‐sEVs^siMDM2^. To confirm whether WPD could be removed from WPD‐sEVs^siMDM2^ by ultracentrifugation, 1 ml rhodamine B labeled WPD in PBS solution at the concentration of 100 µg ml^−1^ was diluted in 100 ml PBS solution and then purified by ultracentrifugation. Next, the ultracentrifuge tube bottom was washed with PBS and detected by a multifunctional microplate reader (Varioskan LUX, Thermo Fisher Scientific, USA).

To further validate whether WPD modification could alter the integrity and contents of sEVs, a series of experiments were performed. First, NFC analysis (N30 NanoFCM, China) was used to detect the modification rate and size distribution of WPD‐sEVs^siMDM2^. Surface charge of unmodified sEVs and WPD‐sEVs^siMDM2^ was detected by a nanoparticle analyzer (DelsaMax Pro, Beckman Coulter, USA). TEM (HitachiH‐7650, Japan) was used for morphology observation of unmodified sEVs and WPD‐sEVs^siMDM2^. To detect the expressions of surface markers in unmodified sEVs and WPD‐sEVs^siMDM2^, the protein samples were collected from MSCs, unmodified sEVs, and WPD‐sEVs^siMDM2^ using the RIPA lysis buffer (Beyotime, China). Western blot analysis were performed as described previously.^[^ ^15^ ^]^ Briefly, after being incubated with 5% BSA for 60 min at room temperature for blocking the nonspecific binding, the polyvinylidene difluoride membranes were incubated with various primary antibodies including CD9 (1:1000), CD63 (1:1000), TSG101 (1:1000), and GM130 (1:1000) at 4 °C overnight. Next, the membranes were incubated with horseradish peroxidase (HRP)‐labeled secondary antibody for 60 min at room temperature, and chemiluminescent signal of each band was visualized by using the ECL detection kit and Bio‐RAD imaging system.

### Stability of sEVs in Vitro

To evaluate the stability of WPD modification, the modification rate and average particle diameter and surface charge of WPD‐sEVs^siMDM2^ were detected after incubation for different time points by NFC analysis and the nanoparticle analyzer. WPD‐sEVs^siMDM2^ or rhodamine B labeled WPD‐sEVs^siMDM2^ were placed in PBS for a 7‐day period (1, 2, 4, and 7 days) at room temperature. Additionally, to explore the time‐dependent stability of WPD‐sEVs^siMDM2^ under physiologically mimic joint microenvironment, WPD‐sEVs^siMDM2^ were incubated with 10 mg ml^−1^ HA or CS at 37 °C for 1, 2, 4, and 7 days. After incubation, WPD‐sEVs^siMDM2^ were isolated by 0.22 µm filtration and serial ultracentrifugation, then the modification rate was detected by NFC.

### Chondrocytes Culture

All procedures in this study were approved by the Independent Ethics Committee of Shanghai Sixth People's Hospital Affiliated to Shanghai Jiao Tong University School of Medicine (Approval Number: 2022‐0611, October 24, 2022, Shanghai, China). Written informed consent was provided from the patients. Human articular chondrocytes were isolated from knee joint cartilage of OA patients. As previously described,^[^ ^15^ ^]^ the harvested articular cartilage was cut into 1 mm^3^ and incubated with 0.25% collagenase II in DMEM‐F12 medium supplemented with 1% Penicillin–Streptomycin for 4 h at 37 °C. After digestion, human chondrocytes were resuspended and filtered through a 40 µm cell filter (Corning, USA) before seeding at a density of 1 × 10^5^ cells ml^−1^ in DMEM‐F12 medium supplemented with 1% Penicillin–Streptomycin and 10% FBS at 37 °C under 5% CO_2_. Chondrocytes at passage 2 were used in the following experiments. To mimic the senescent phenotype of chondrocytes in OA cartilage, a DNA‐damaging chemical agent doxorubicin at the concentration of 1 µM was used to treat the human chondrocytes once every 2 days for 14 days,^[^ ^19b^ ^]^ and then treated with sEVs (1 × 10^10^ particles ml^−1^), sEVs^siMDM2^ (1 × 10^10^ particles ml^−1^), or WPD‐sEVs^siMDM2^ (1 × 10^10^ particles ml^−1^) for 7 days.

### Cellular Uptake of sEVs

Lipid dye DiO was used to label sEVs according to previously published article.^[^ ^15^ ^]^ Briefly, unmodified sEVs (1 × 10^10^ particles ml^−1^) and WPD‐sEVs^siMDM2^ (1 × 10^10^ particles ml^−1^) were incubated with 10 µM DiO for 30 min at 37 °C in the dark. Subsequently, the suspension was filtered by 0.22 µm membrane and centrifuged at 100,000×*g* for 140 min to remove the free DiO. Next, the purified DiO‐labeled sEVs and WPD‐sEVs^siMDM2^ were incubated with chondrocytes for 12 h at 37 °C under 5% CO_2_. After incubation, the chondrocytes were fixed with 4% PFA for 30 min and then stained with DAPI for 5 min. Images in each group were observed by the confocal microscopy (Leica Microsystems, Germany). In addition, the DiO staining of PBS only group were treated with the same isolation procedure to exclude the possibility of the nonspecific labelling. The results were also analyzed by the CytoFLEX flow cytometry (Beckman Coulter, USA) and the mean fluorescence intensity of DiO‐positive unmodified sEVs and WPD‐sEVs^siMDM2^ were evaluated.

### CCK8 Assay

A Cell Counting kit‐8 (CCK8) kit (Dojindo, Japan) was applied to evaluate the chondrocyte proliferation according to manufacturers’ instruction. As described previously,^[^ ^15^, ^28^ ^]^ human chondrocytes were seeded into 96‐well plate at 3000 cells per well. After being incubated with WPD, unmodified sEVs, or WPD‐sEVs^siMDM2^ for different time points, the absorbance in each group was detected at 450 nm by a multifunctional microplate reader (Varioskan LUX, Thermo Fisher Scientific, USA).

### PCR Analysis

Total RNA was isolated from human chondrocytes by using the RNeasy kit following the manufacturer's instruction (Qiagen, Germany). Total RNA from human cartilage explants was isolated by using the TRIzol reagent (0.1 ml TRIzol g^−1^ tissue, Invitrogen, USA), followed by the chloroform and phenol acid extraction. The concentration of total RNA was measured at OD260. The complementary DNA (cDNA) was obtained by extracted RNA by reverse transcription using the PrimeScriptTM RT reagent Kit (RR047A, TaKaRa, Japan). SYBR Green (RR420A, TaKaRa, Japan) was applied to proceed with PCR reactions following the manufacturer's recommendation and human gene *β‐actin* was set as the housekeeping gene. The relative gene expressions of the mRNAs were analyzed by using the 2^−ΔΔCT^ method.^[^ ^29^ ^]^ All primers used for PCR analysis were purchased from Sangon Biotech (Shanghai, China) and are listed in Table S5 (Supporting Information).

### Western Blot Analysis

The protein samples were collected from chondrocyte lysates using RIPA solution. The Western blot analysis was performed as described previously,^[^ ^30^ ^]^ after blocked with 5% BSA for 60 min at room temperature to block the unspecific absorption and then the PVDF membranes were incubated with various primary antibodies overnight at 4 °C. Subsequently, the membranes were incubated with horseradish peroxidase (HRP)‐labeled secondary antibody at room temperature for 60 min. The chemiluminescent signals were visualized by the ECL Western Blot detection kit and Bio‐RAD imaging system.

### Immunofluorescence Staining

Immunofluorescence staining was performed to detect the expressions of P16^INK4a^, γH2AX, and MDM2 in chondrocytes. The chondrocytes were fixed in 4% PFA and immersed in 5% BSA for 1 h at 37 °C. Then, the cells were incubated with different primary antibodies including P16^INK4a^ (1:100), γH2AX (1:100), P21 (1:100), and MDM2 (1:100) at 4 °C overnight. Next, the secondary antibody (1:200) with fluorescence was added to the cells for 1 h at room temperature in the dark. After incubation, cells were stained with DAPI for 5 min and then observed using a confocal microscope (Leica Microsystems, Germany).

### TUNEL Staining

After different treatments, the apoptosis rates of chondrocytes in all groups were detected using a TUNEL staining kit (Beyotime, China) according to the protocol.

### SA‐β‐Gal Staining

SA‐β‐gal staining was performed as previously described.^[^ ^31^ ^]^ The cell senescence β‐galactosidase staining kit (Beyotime, China) was applied according to the manufacturer's instruction. Senescent chondrocytes were identified as blue‐stained cells under light microscopy (Leica Microsystems, Germany). Total cells and SA‐β‐Gal positive cells were counted in three random fields per culture dish to analyzed the percentage of SA‐β‐gal positive cells.

### Human Articular Cartilage Harvest and Culture

Two types of human articular cartilage specimens were collected in this study. OA‐affected articular cartilage (OA cartilage) and non‐OA affected articular cartilage (normal cartilage) were both collected from six OA patients (mean ± S.D. age, 75.17 ± 6.62 years old; range, 67–85 years old) undergoing total knee joint replacement. For ex vivo culture of OA cartilage explants, explants were harvested from OA‐affected area or non‐OA affected area of articular cartilage using biopsy punch (5 mm in diameter and 1 mm in thickness) and washed with PBS supplemented with 1% Penicillin–Streptomycin for three times. Next, the cartilage explants were cultured in DMEM‐F12 medium supplemented with 10% FBS and 1% Penicillin–Streptomycin in a 48‐well‐plate. For the treatments on OA cartilage explants, sEVs (1 × 10^10^ particles ml^−1^), sEVs^siMDM2^ (1 × 10^10^ particles ml^−1^), or WPD‐sEVs^siMDM2^ (1 × 10^10^ particles ml^−1^) were applied for 7 days.

### Articular Cartilage Penetration Assay

Articular cartilage explants (Φ 5 mm, 1 mm thickness) were harvested from the knee joints of OA patients by using an electric drill. After extraction, the cartilage explants were washed with PBS containing 1% Penicillin–Streptomycin. In order to evaluate the cartilage uptake and penetration capacity of unmodified sEVs and WPD‐sEVs^siMDM2^. Lipophilic fluorescence dye DiO was used to label unmodified sEVs and WPD‐sEVs^siMDM2^. The cartilage uptake efficiency was measured as the percentage of sEVs from the bath solution to the cartilage explants. The cartilage explants were immersed in 200 µl PBS containing DiO labeled unmodified sEVs and WPD‐sEVs^siMDM2^ in a 96‐well plate at 37 °C for 1, 3, and 7 days. After that, the cartilage explants were removed, and the DiO fluorescent intensity was recorded by the multifunctional microplate reader (Varioskan LUX, Thermo Fisher Scientific, USA).

Next, a self‐designed one‐way transport teflon mould was applied to assess the cartilage penetration ability of unmodified sEVs and WPD‐sEVs^siMDM2^ as described previously.^[^ ^15^ ^]^ Briefly, the freshly harvested cartilage explants were stuck into the hole in the removable baffle which divided the mould to two chambers. 200 µl DiO labeled unmodified sEVs or WPD‐sEVs^siMDM2^ were added to one side chamber of the mould, while 200 µl sterilized PBS was added to the other chamber of the mould. After penetration for different time points, the cartilage explants were immersed with OCT glue and sectioned to 5 µm slides by a vibrating microtome (Leica, Germany) followed by an immediate microscopy observation (Leica Microsystems, Germany).

### Joint Retention of sEVs In Vivo

All animal experimental procedures were approved by the Animal Research Committee of Shanghai Sixth People's Hospital Affiliated to Shanghai Jiao Tong University School of Medicine (Approval Number: 2022‐0611, October 24, 2022, Shanghai, China). The joint retention capacity of unmodified sEVs or WPD‐sEVs^siMDM2^ in mouse was detected over a period of 35 days by using an IVIS Spectrum imaging system (PerkinElmer, USA). For in vivo imaging study, a near‐infrared fluorescence lipid dye DiR was used to label unmodified sEVs and WPD‐sEVs^siMDM2^. Briefly, 10 µl DiR‐labeled unmodified sEVs or WPD‐sEVs^siMDM2^ (1 × 10^10^ particles ml^−1^) was injected to the mouse knee joint cavity. After intra‐articular injection, images of each mouse joint were recorded by the IVIS Spectrum imaging system at different time points (0, 1, 3, 7, 14, 28, and 35 days). The data of total radiant efficiency within the region of each knee joint was calculated and presented along with time‐based on previous studies.^[^ ^15^, ^32^ ^]^

### Animal OA Models

To establish PTOA model, 8‐week‐old male mice were subjected to sham surgery or ACLT surgery as described previously.^[^ ^19^, ^20^ ^]^ Briefly, the right joint capsule was opened and the anterior cruciate ligament was transected to destabilize the right knee joint in ACLT group mice, whereas the joint capsule was opened without any other operation in sham group mice. For the administration of different treatments in ACLT‐induced PTOA mice, 10 µl PBS, sEVs (1 × 10^10^ particles ml^−1^), sEVs^siMDM2^ (1 × 10^10^ particles ml^−1^), or WPD‐sEVs^siMDM2^ (1 × 10^10^ particles ml^−1^) was intra‐articularly injected into the right knee joint cavity of mice once every four weeks by using a 10 µl micro syringe (Hamilton, USA) four weeks after surgery. All mice were euthanized eight weeks after different treatments. For the administration of different treatments in naturally aged OA mice, 10 µl PBS, sEVs (1 × 10^10^ particles ml^−1^), sEVs^siMDM2^ (1 × 10^10^ particles ml^−1^), or WPD‐sEVs^siMDM2^ (1 × 10^10^ particles ml^−1^) was intra‐articularly injected into the right knee joint cavity of 12‐month‐old mice once a month. Mice were euthanized after 8 months treatments. 8‐month‐old mice were used as the young control group.

### Histological Analysis

The harvested knee joints in each group were fixed immediately in 4% PFA for 24 h, dehydrated using the gradient alcohol, vitrified with dimethyl benzene, and embedded in the paraffin. The paraffin sections (thickness: 5 µm) were deparaffinized in xylene, hydrated with gradient ethanol, and stained with Safranin O and H&E solutions. Additionally, the score of cartilage loss and destruction in each group was evaluated by using the mouse OARSI scoring system,^[^ ^33^ ^]^ and the synovial inflammation was also assessed according to previously published article.^[^ ^19b^ ^]^ In addition, subchondral bone was evaluated by a method which was published previously.^[^ ^34^ ^]^

### Immunohistochemical Analysis

For evaluation of the immunoreactivity to collagen II, SOX9, MMP13, P16^INK4a^, P21, IL6, HMGB1, TNFα, MDM2, P53, and p‐P53, histological sections were deparaffinized and then incubated with 0.3% hydrogen peroxide at room temperature for 30 min. Primary antibodies used in this study were listed in Table S3 (Supporting Information). Subsequently, the enzyme‐activated antigen retrieval (0.1% trypsin) for each section was performed for 10 min at room temperature. Then, all sections were blocked with 5% BSA for 1 h at room temperature and incubated with primary antibodies overnight at 4 °C. Next, appropriate HRP‐labeled secondary antibodies (1:200) were applied to incubation with the sections for 1 h at room temperature. After that, all sections were observed and analyzed under a light microscopy (Leica Microsystems, Germany).

### ELISA Analysis

The cartilage tissues were also harvested and homogenized in 1 ml PBS.^[^ ^35^ ^]^ After centrifugation at 10 000×*g* for 15 min at 4 °C, the supernatants in different groups were used for examining the expressions of inflammatory mediators IL6 and TNFα by the corresponding ELISA kits. The microplate reader was applied to measure the absorbance at 450 nm, and the final presented data for each group was normalized with the control group.

### Statistical Analysis

Statistical analysis was performed by using GraphPad Prism software 8. All the experimental results were expressed as the mean ± standard deviation (SD). Differences between groups were evaluated by the two‐sided Student's *t*‐test (for comparison of two groups) or the one‐way analysis of variance (ANOVA) with Tukey's test (for comparison of more than two groups). Data quantified based on ordinal grading systems including the OARSI grade, synovial inflammation score, and the subchondral bone score, whose data points are not continuous and do not follow a normal distribution, were analyzed using nonparametric statistical method based on Mann–Whitney *U* test was used. Statistical significance level was defined as *P* < 0.05 for hypothesis testing.

## Conflict of Interest

The authors declare no conflict of interest.

## Author Contributions

K.F., J.S.L., and L.Z.G. contributed equally to this work. K.F., X.T.X., Y.W., and Q.L. designed the study. K.F., J.S.L., L.Z.G., T.Y., Z.S.C., Q.L., and X.T.X. performed the experiments. K.F., J.S.L., and L.Z.G. performed the material development. K.F., L.Z.G., J.S.L., Z.S.C., and T.Y. contributed to the data analysis and interpretation. K.F., Y.W., Q.L., and X.T.X. wrote the manuscript. All authors have read and agreed to the published version of the manuscript.

## Supporting information



Supporting Information

## Data Availability

The data that support the findings of this study are available from the corresponding author upon reasonable request.
