# Supplementary material for: Engineered MSC‐sEVs as a Versatile Nanoplatform for Enhanced Osteoarthritis Treatment via Targeted Elimination of Senescent Chondrocytes and Maintenance of Cartilage Matrix Metabolic Homeostasis
Source: Adv Sci (Weinh). 2025 Jan 4;12(8):2413759. doi: 10.1002/advs.202413759 (PMC11848604; doi:10.1002/advs.202413759)
Supplement: Supplementary file 1 — Supporting Information [file ADVS-12-2413759-s001.docx]

**Supporting Information for**

**Engineered MSC-sEVs as A Versatile Nanoplatform for Enhanced Osteoarthritis Treatment via Targeted Elimination of Senescent Chondrocytes and Maintenance of Cartilage Matrix Metabolic Homeostasis**

Kai Feng, Jiashuo Liu, Liangzhi Gong, Teng Ye, Zhengsheng Chen, Yang Wang,* Qing Li,* and Xuetao Xie*

Institute of Microsurgery on Extremities, Department of Orthopedic Surgery, Shanghai Sixth People’s Hospital Affiliated to Shanghai Jiao Tong University School of Medicine, Shanghai 200233, China

E-mail: xuetaoxie@163.com; liqing_236@aliyun.com; wangyang63@sjtu.edu.cn

**Figure S1**

**
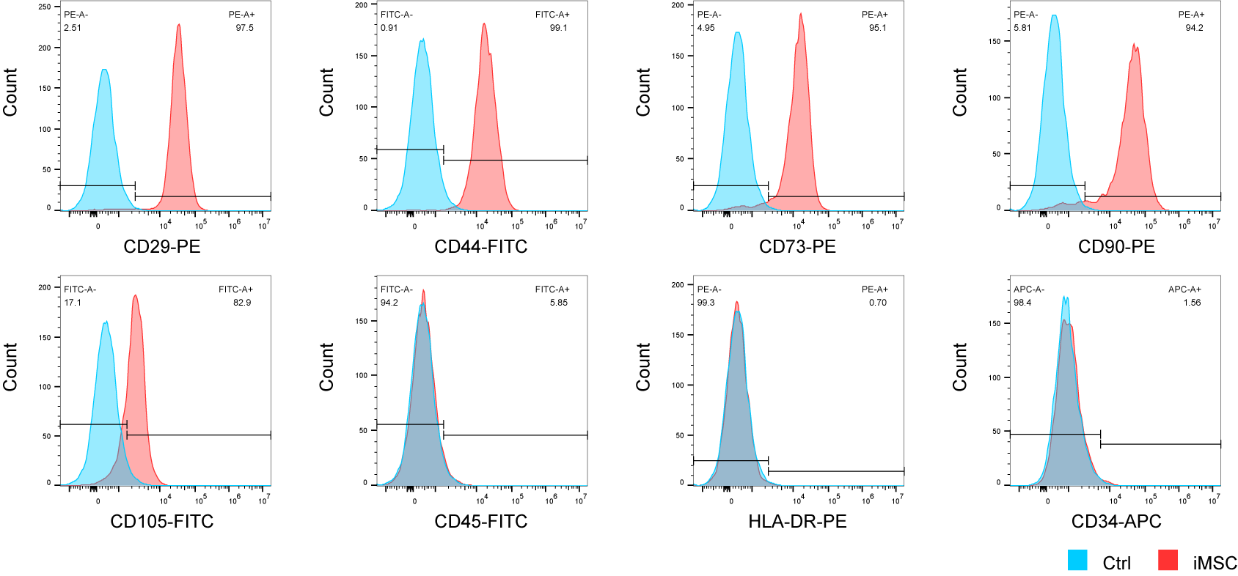
**

**Figure S1.** Identification of induced pluripotent stem cell-derived mesenchymal stem cells (iMSCs) by flow cytometry analysis. iMSCs were positive for CD29, CD44, CD73, CD90, and CD105, and were negative for CD45, HLA-DR, and CD34.

**Figure S2**


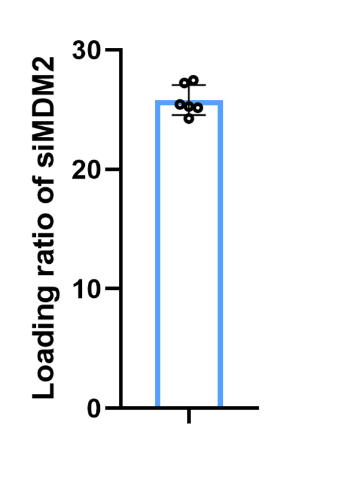


**Figure S2.** The loading rate of siMDM2 in MSC-sEVs (n = 6).

**Figure S3**

**
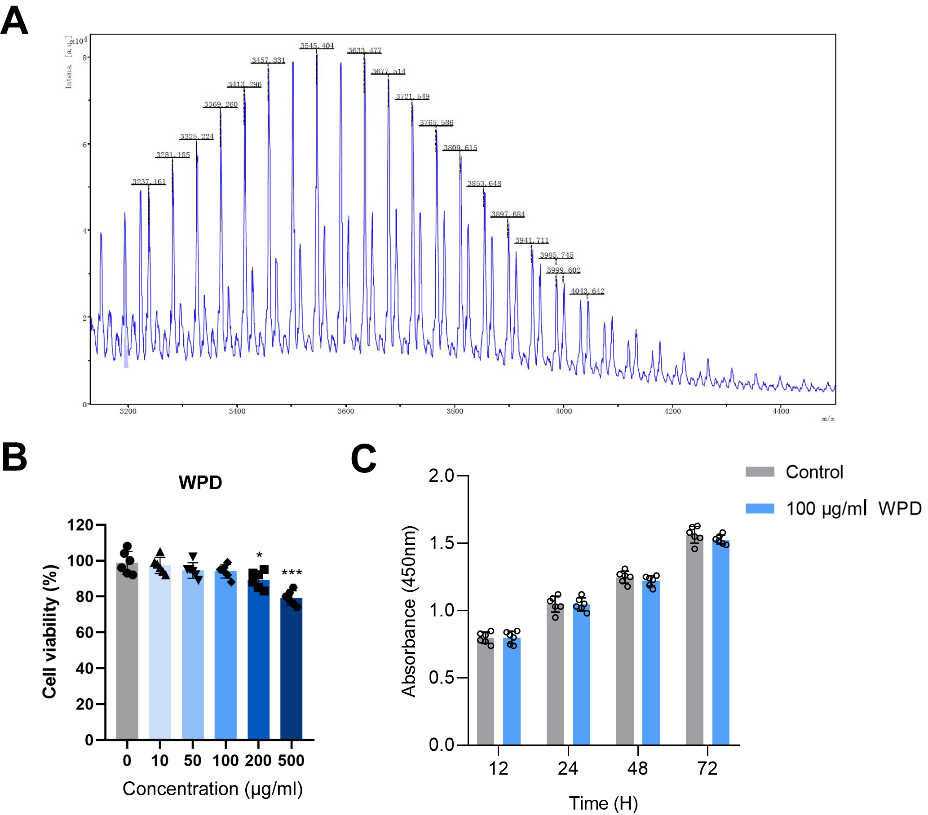
**

**Figure S3.** The cytocompatible of WPD on human chondrocytes. A) MOLDI-TOF analysis of WPD peptide. B) WPD at different concentrations (0 – 500 μg/ml) on chondrocytes (n = 6). C) WPD at the concentration of 100 μg/ml on human chondrocytes for different time points (n = 6). Data are represented as mean ± SD. *P < 0.05, ***P < 0.001.

**Figure S4**


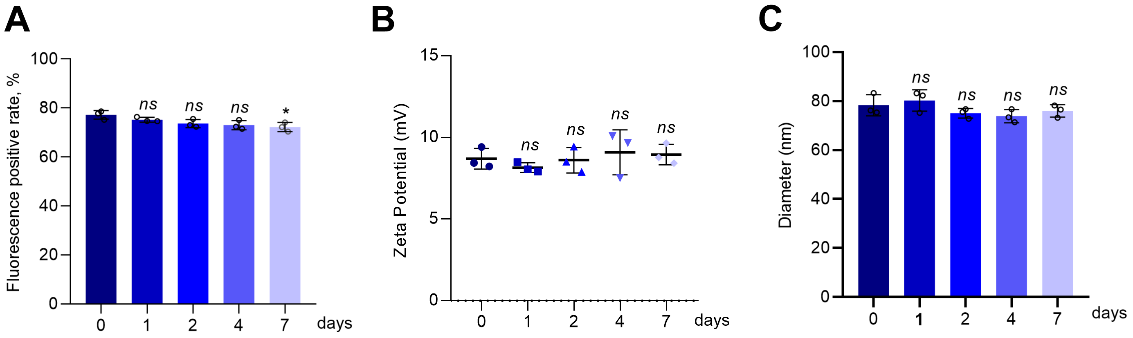


**Figure S4.** The stability of WPD modification on MSC-sEVs in PBS solution. A) The change of WPD modification rate in a 7-day period (n = 3). B) The zeta potential of WPD-sEVs^siMDM2^ was detected in a 7-day period (n = 3). C) The average particle diameter of WPD-sEVs^siMDM2^ was in a 7-day period (n = 3). Data are represented as mean ± SD. *P < 0.05, *ns*, not significant.

**Figure S5**


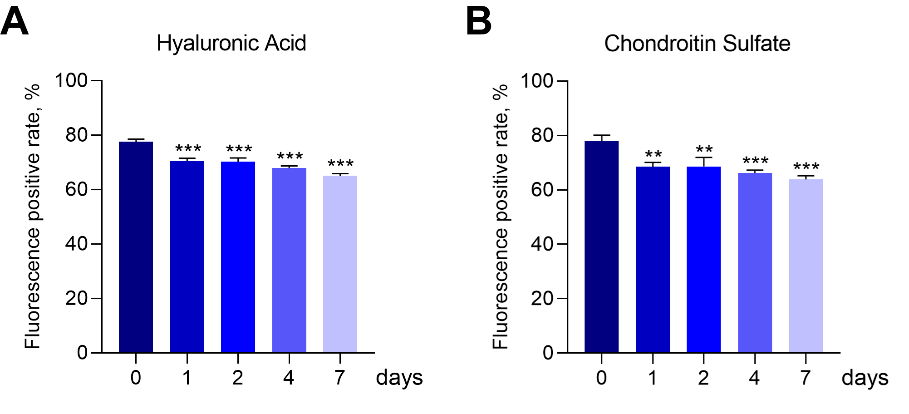


**Figure S5.** The stability of WPD modification on MSC-sEVs in hyaluronic acid (HA) or chondroitin sulfate (CS) solution. A) WPD modification stability under the interference of 10 mg/ml HA solution in a 7-day period (n = 3). B) WPD modification stability under the interference of 10 mg/ml CS solution in a 7-day period (n = 3). Data are represented as mean ± SD. **P < 0.01, ***P < 0.001.

**Figure S6**

**
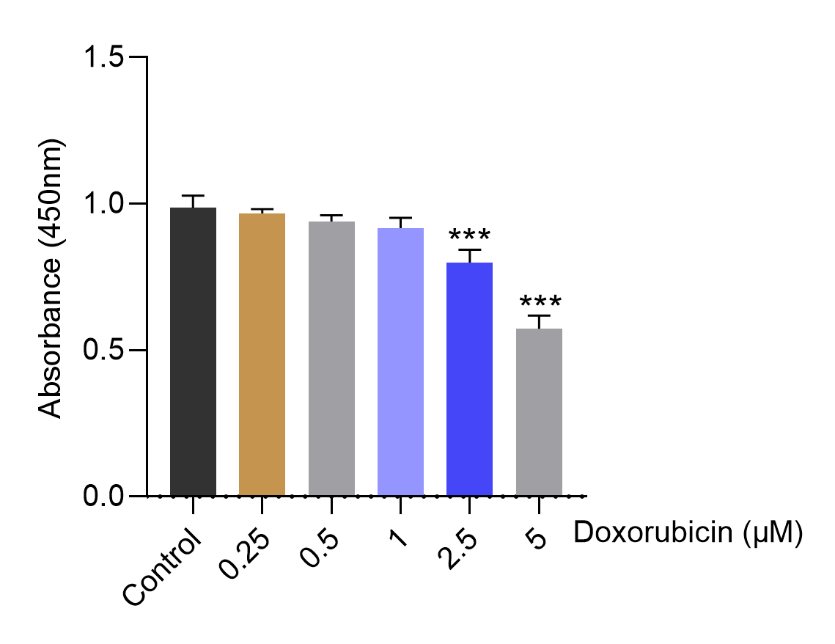
**

**Figure S6.** The viability of chondrocytes after treatment of doxorubicin at different concentrations (n = 3). Data are represented as mean ± SD. *P < 0.05, *ns*, not significant.

**Figure S7**

**
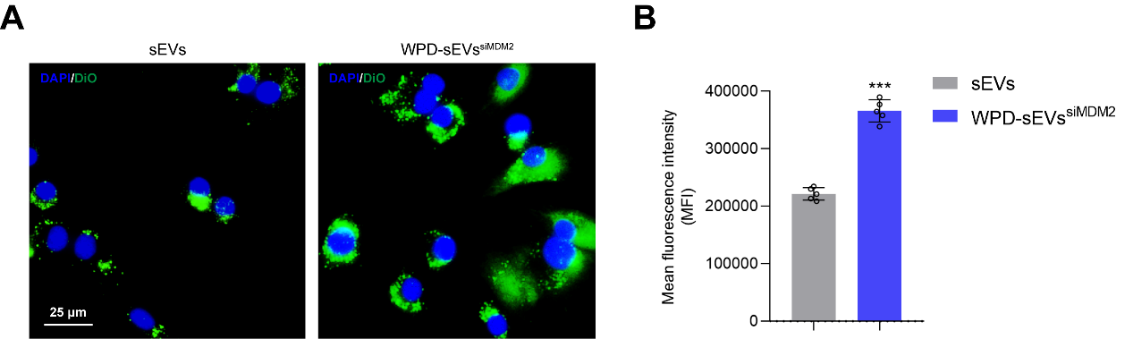
**

**Figure S7.** Cellular uptake efficiency of sEVs and WPD-sEVs^siMDM2^ in chondrocytes. A) Representative images of the chondrocyte uptake of DiO (Green) labelled unmodified sEVs or WPD-sEVs^siMDM2^. Scale bar: 50 μm. B) Mean fluorescence intensity (MFI) of DiO-labelled sEVs and WPD-sEVs^siMDM2^ in each group (n = 5). Data are represented as mean ± SD. ***P < 0.001.

**Figure S8**

**
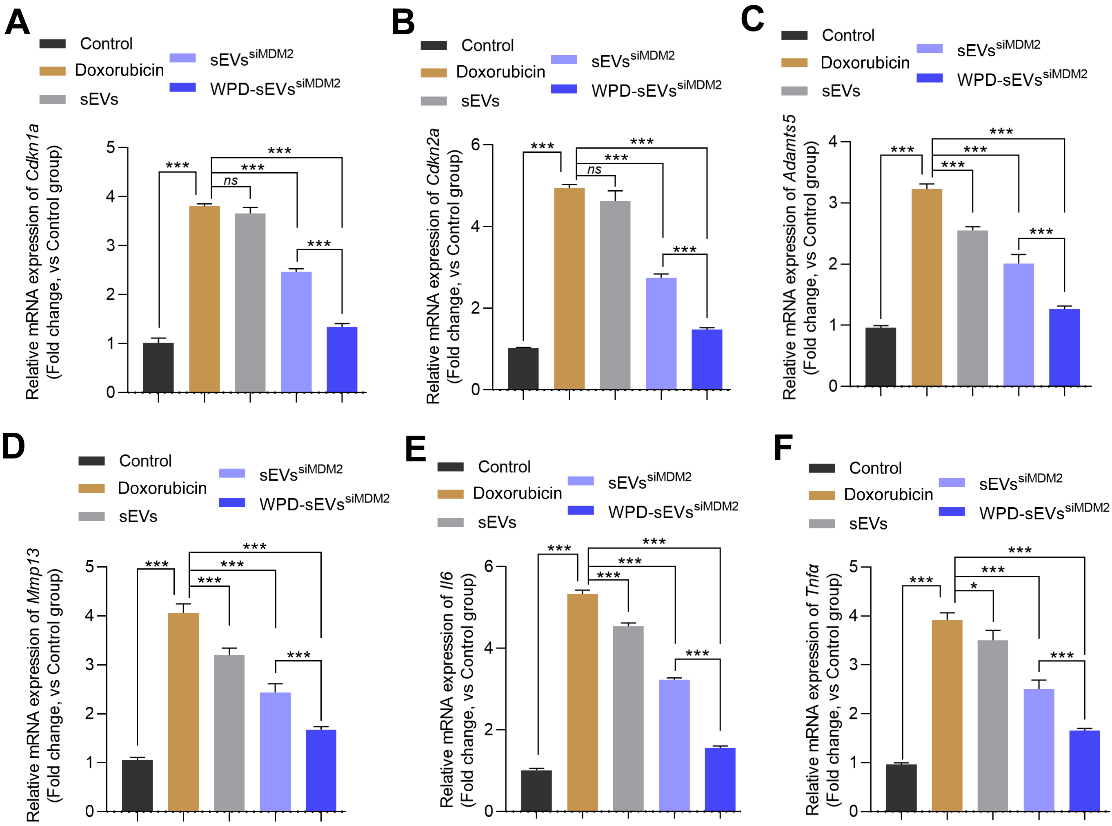
**

**Figure S8.** PCR analysis of senescence and SASP markers in chondrocytes after different treatments. A-B) PCR analysis of senescence-related markers *Cdkn1a* and *Cdkn2a* gene expressions in chondrocytes after different treatments (n = 3). C-D) PCR analysis of cartilage matrix-related genes *Adamts5* and *Mmp13* expressions in chondrocytes after different treatments (n = 3). E-F) PCR analysis of SASP factors *Il6* and *Tnfα* gene expressions in chondrocytes after different treatments (n = 3). Data are represented as mean ± SD. ***P < 0.001, *ns*, not significant.

**Figure S9**


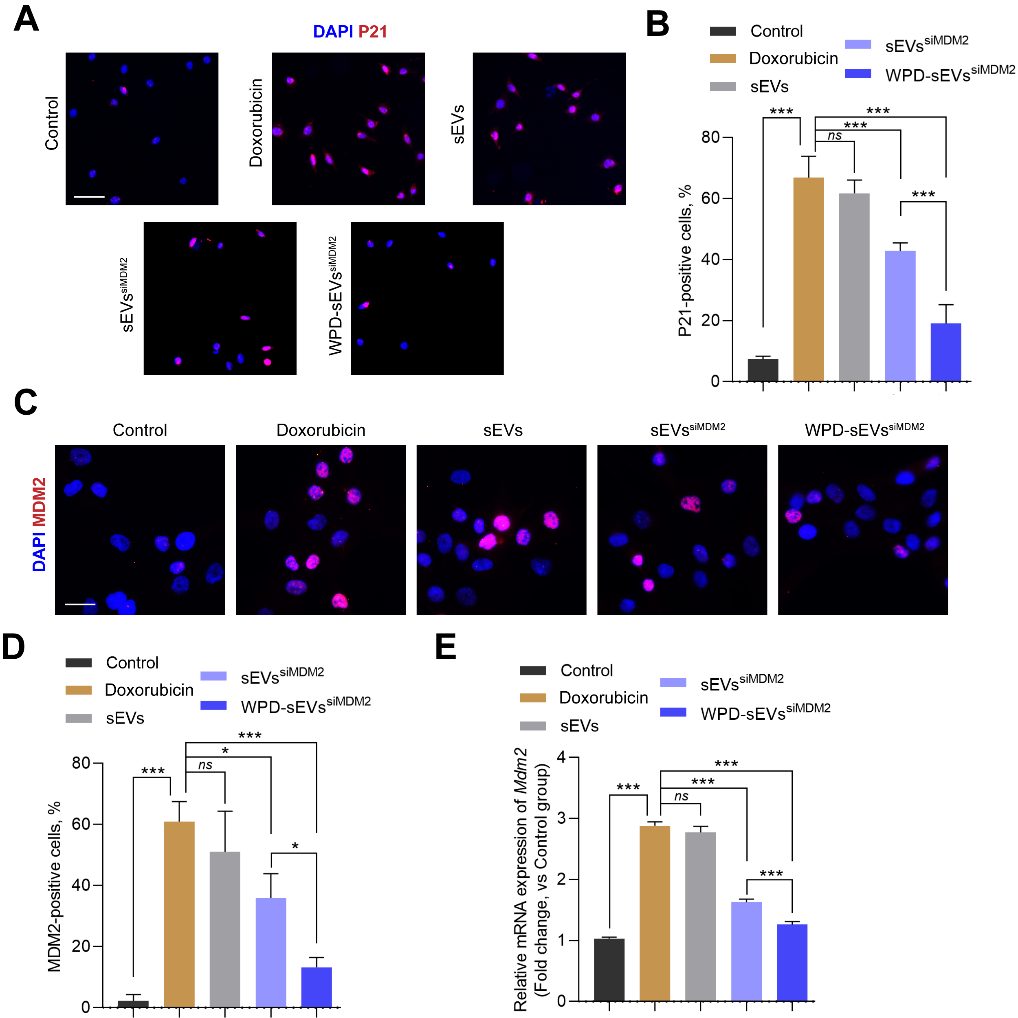


**Figure S9.** The effect of WPD-sEVs^siMDM2^ on P21 and MDM2 expression in chondrocytes. A-B) Immunofluorescence staining and statistical evaluation for P21 in chondrocytes. Scale bar: 100 μm. C-D) Immunofluorescence staining and statistical evaluation for MDM2 in chondrocytes. Scale bar: 100 μm. E) PCR analysis of *Mdm2* gene expression in chondrocytes after different treatments (n = 3). Data are represented as mean ± SD. *P < 0.05 and ***P < 0.001, *ns*, not significant.

**Figure S10**

**
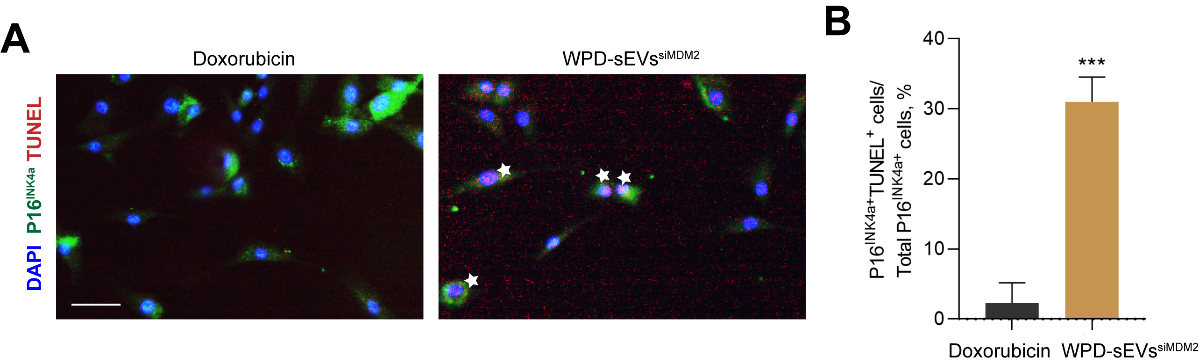
**

**Figure S10.** Immunofluorescence co-localization A) and quantitative analysis B) of senescence-related marker P16^INK4a^ and apoptosis-related marker TUNEL in senescent chondrocytes after treatment of WPD-sEVs^siMDM2^. Scale bar: 100 μm. Data are represented as mean ± SD. ***P < 0.001.

**Figure S11**

**
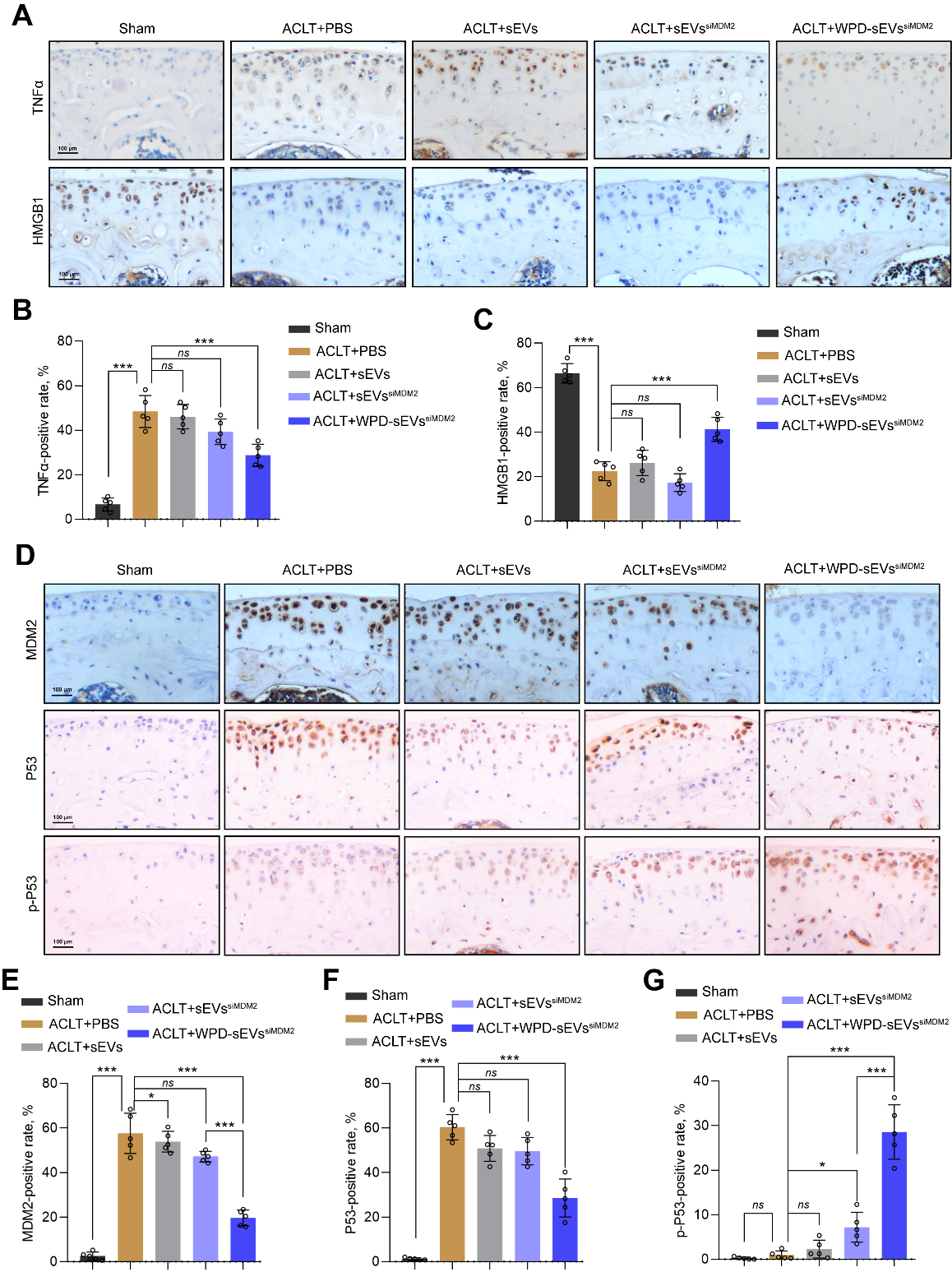
**

**Figure S11.** Immunohistochemical staining for senescence markers in cartilage of PTOA mice.

A-C) Representative images and quantitative analysis of immunohistochemical staining for TNFα and HMGB1 in mice articular cartilage (n = 5). Scale bar: 100 μm. D-G) Representative images and quantitative analysis of immunohistochemical staining for MDM2, P53 and p-P53 in mice articular cartilage (n = 5). Scale bar: 100 μm. Data are represented as mean ± SD. *P < 0.05 and ***P < 0.001, *ns*, not significant.

**Figure S12**

**
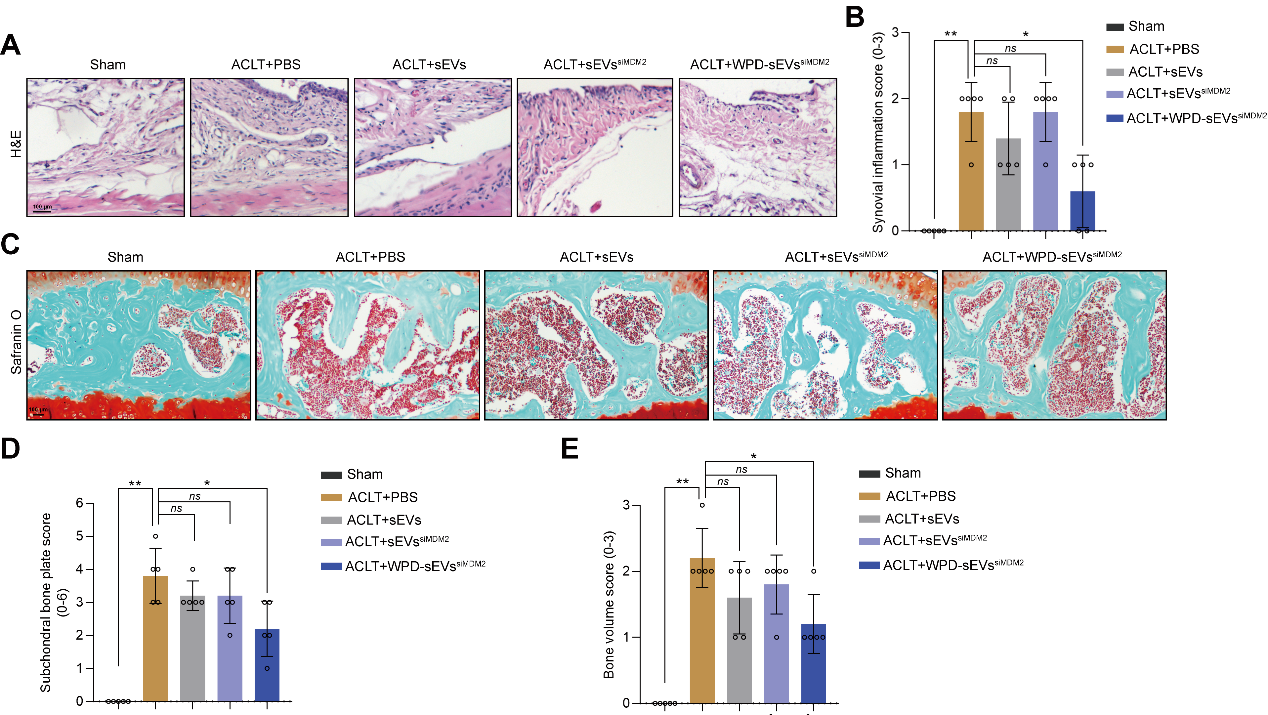
**

**Figure S12.** Evaluation of joint synovium inflammation and subchondral bone in PTOA mice. A) Representative micrographs of H&E staining for synovium in the knee joint. Scale bar: 100 μm. B) Synovial inflammation score after different treatments (n = 5). C) Representative micrographs of Safranin O staining for subchondral bone in the knee joint. Scale bar: 100 μm. D-E) Subchondral bone plate score and bone volume score analysis in mice knee joint after different treatments (n = 5). Data are represented as mean ± SD. *P < 0.05 and **P < 0.01, *ns*, not significant.

**Figure S13**

**
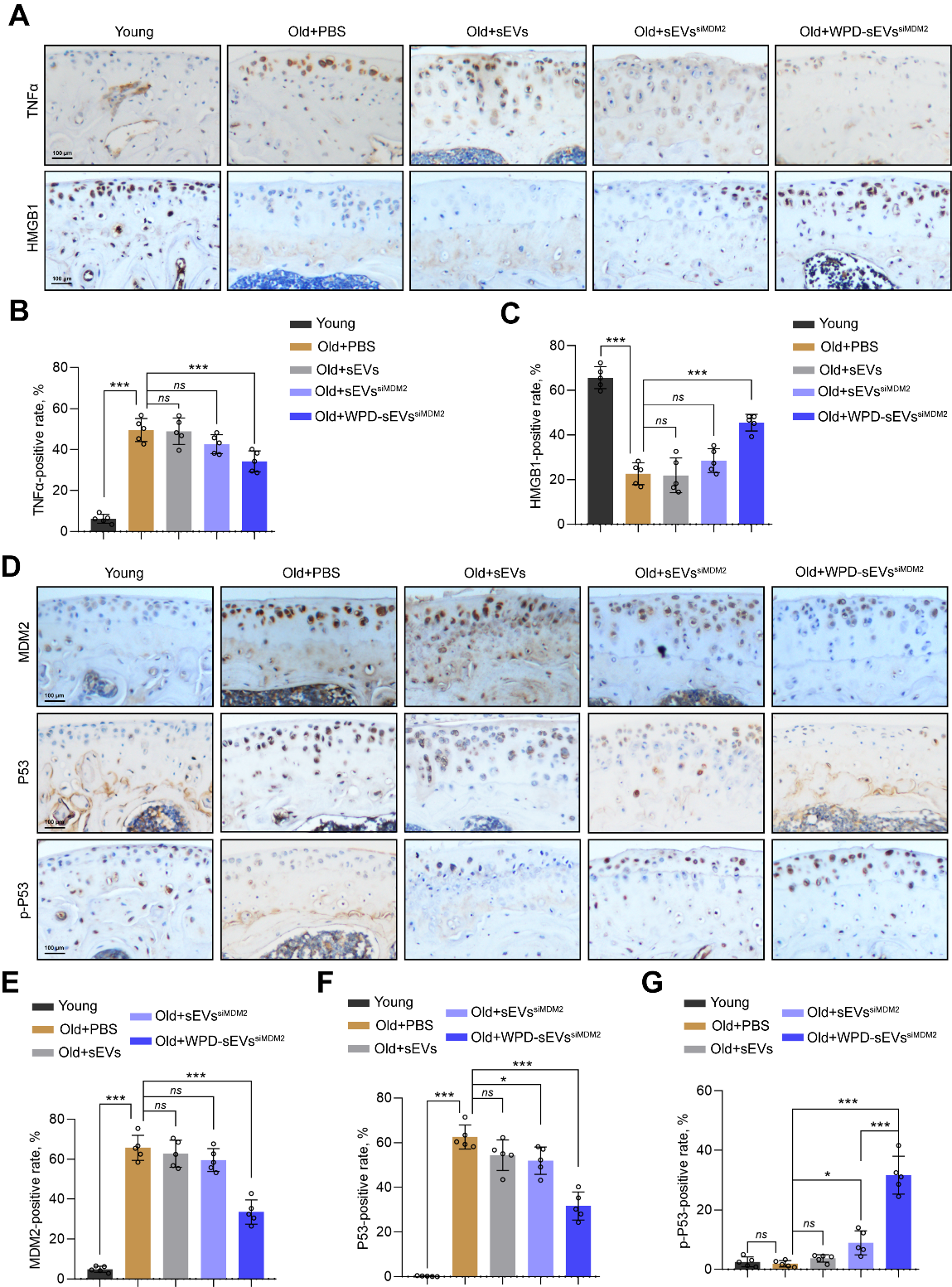
**

**Figure S13.** Immunohistochemical staining for senescence markers in articular cartilage of naturally aged mice. A-C) Representative images and quantitative analysis of immunohistochemical staining for TNFα and HMGB1 in mice cartilage (n = 5). Scale bar: 100 μm. D-G) Representative images and quantitative analysis of immunohistochemical staining for MDM2, P53 and p-P53 in mice cartilage (n = 5). Scale bar: 100 μm. Data are represented as mean ± SD. *P < 0.05 and ***P < 0.001, *ns*, not significant.

**Figure S14**


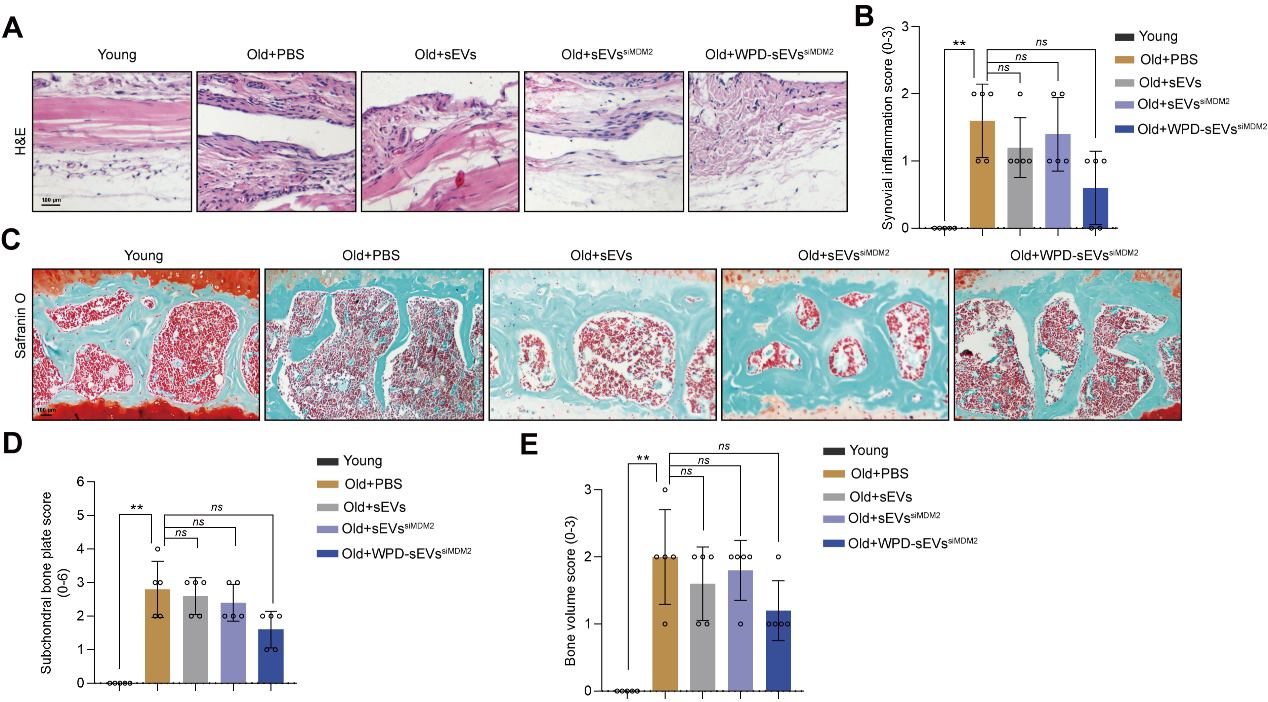


**Figure S14.** Evaluation of joint synovium inflammation and subchondral bone in naturally aged mice. A) Representative micrographs of H&E staining for synovium in the knee joint. Scale bar: 100 μm. B) Synovial inflammation score after different treatments (n = 5). C) Representative micrographs of Safranin O staining for subchondral bone in the knee joint. Scale bar: 100 μm. D-E) Subchondral bone plate score and bone volume score analysis in mice knee joint after different treatments (n = 5). Data are represented as mean ± SD. **P < 0.01, *ns*, not significant.

**Table S1.** Data of modification rate of WPD to MSC-sEVs in10 mg/ml HA or CS.

|  |  | **WPD-sEVs^siMDM2^** | |
| --- | --- | --- | --- |
|  | Time/d | Mean% | SD |
| HA  (10 mg/ml) | 0 | 77.50 | 0.86 |
|  | 1 | 70.47 | 0.90 |
|  | 2 | 70.17 | 1.19 |
|  | 4 | 67.83 | 0.74 |
|  | 7 | 65.13 | 0.57 |
| CS  (10 mg/ml) | 0 | 77.90 | 1.84 |
|  | 1 | 68.57 | 1.24 |
|  | 2 | 68.63 | 2.74 |
|  | 4 | 66.20 | 0.90 |
|  | 7 | 63.83 | 1.13 |

**Table S2.** Data of cartilage uptake percentage.

|  |  | OA articular cartilage | |
| --- | --- | --- | --- |
|  | Time/d | Mean% | SD |
| WPD-sEVs^siMDM2^  (10^10^ particles/ml) | 0 |  |  |
|  | 1 | 47.33 | 4.50 |
|  | 3 | 55.67 | 2.87 |
|  | 7 | 64.00 | 2.45 |
| MSC-sEVs  （10^10^ particles/ml） | 0 |  |  |
|  | 1 | 12.33 | 2.05 |
|  | 3 | 14.67 | 1.25 |
|  | 7 | 20.33 | 1.25 |

**Table S3.** The primary and secondary antibodies used in our study.

| Antibody | Catalog# | Company |
| --- | --- | --- |
| CD9 | ab236630 | Abcam |
| CD63 | ab134045 | Abcam |
| TSG101 | ab133586 | Abcam |
| GM130 | ab52649 | Abcam |
| MDM2 | ab259265 | Abcam |
| MDM2 | ab226939 | Abcam |
| Collagen Ⅱ | ab34712 | Abcam |
| MMP13 | ab219620 | Abcam |
| SOX9 | ab185966 | Abcam |
| P16^INK4a^ | ab270058 | Abcam |
| P16^INK4a^ | ab241543 | Abcam |
| P21 | Ab109199 | Abcam |
| P53 | ab131442 | Abcam |
| p-P53 | ab33889 | Abcam |
| IL6 | ab290735 | Abcam |
| γH2AX | ab229914 | Abcam |
| HMGB1 | ab92310 | Abcam |
| TNFα | ab183218 | Abcam |
| Anti-Rabbit IgG H&L  (HRP Conjugated) | ab6721 | Abcam |
| Anti-Rabbit IgG H&L  (Alexa Fluor® 488) | ab150077 | Abcam |
| Anti-Rabbit IgG H&L  (Alexa Fluor® 594) | ab150080 | Abcam |

**Table S4.** Sequences of human siMDM2.

| Product | Forward primer (5’-3’) | Reverse primer (5’-3’) |
| --- | --- | --- |
| hs-GAPDH | GUGGAUAUUGUUGCCAUCATT | UGAUGGCAACAAUAUCCACTT |
| FAM-NC | UUCUCCGAACGUGUCACGUTT | ACGUGACACGUUCGGAGAATT-FAM |
| NC | UUCUCCGAACGUGUCACGUTT | ACGUGACACGUUCGGAGAATT |
| hs-mdm2-siRNA | GGAACUUGGUAGUAGUCAATT | UUGACUACUACCAAGUUCCTT |
| FAM-hs-mdm2-siRNA | GGAACUUGGUAGUAGUCAATT | UUGACUACUACCAAGUUCCTT-FAM |

**Table S5.** Human PCR primer sequences.

| Gene | Forward primer (5’-3’) | Reverse primer (5’-3’) |
| --- | --- | --- |
| *Col2a1* | GGAGCAGCAAGAGCAAGGAGAAG | TCATCTGGACGTTGGCAGTGTTG |
| *Acan* | ACGGCTTCTGGAGACAGGACTG | CTGGGATGCTGGTGCTGATGAC |
| *Mdm2* | GAGCCTCCAATGAGAGCAACTTGAG | GCTGCCATGTGACCTAAGAGAAGAC |
| *Cdkn1a* | TCCAGCGACCTTCCTCATCCAC | TCCATAGCCTCTACTGCCACCATC |
| *Cdkn2a* | TAATAGCACCTCCTCCGAGCACTC | ACCCTGTCCCTCAAATCCTCTGG |
| *Il6* | GACAGCCACTCACCTCTTCAGAAC | GCCTCTTTGCTGCTTTCACACATG |
| *Tnfα* | AGGCAATAGGTTTTGAGGGCCAT | TCCTCCCTGCTCCGATTCCG |
| *Mmp13* | GCGGGAATCCTGAAGGAGAATGC | TCAAGTTTGCCAGTCACCTCTAAGC |
| *Adamts5* | GACCGATGGCACTGAATGTAGGC | TCTCCTCCACATACTCCGCACTTG |
| *β-actin* | GAAGATCAAGATCATTGCTCCT | TGGAAGGTGGACAGTGAG |
